# Advances in Zebrafish as a Comprehensive Model of Mental Disorders

**DOI:** 10.1155/2023/6663141

**Published:** 2023-06-20

**Authors:** Lei Wang, Fan Liu, Yimeng Fang, Jiahui Ma, Jiawei Wang, Linkai Qu, Qinsi Yang, Wei Wu, Libo Jin, Da Sun

**Affiliations:** ^1^Institute of Life Sciences & Biomedical Collaborative Innovation Center of Zhejiang Province, Wenzhou University, Wenzhou 325000, China; ^2^Department of Applied Biology and Chemical Technology, The Hong Kong Polytechnic University, Hung Hom, Hong Kong SAR 999077, China; ^3^Wenzhou Institute, University of Chinese Academy of Sciences, Wenzhou 325000, China; ^4^Key Laboratory for Biorheological Science and Technology of Ministry of Education, State and Local Joint Engineering Laboratory for Vascular Implants, Bioengineering College of Chongqing University, Chongqing 400030, China; ^5^Wenzhou City and Wenzhou OuTai Medical Laboratory Co., Ltd. Joint Doctoral Innovation Station, Wenzhou Association for Science and Technology, Wenzhou 325000, China

## Abstract

As an important part in international disease, mental disorders seriously damage human health and social stability, which show the complex pathogenesis and increasing incidence year by year. In order to analyze the pathogenesis of mental disorders as soon as possible and to look for the targeted drug treatment for psychiatric diseases, a more reasonable animal model is imperious demands. Benefiting from its high homology with the human genome, its brain tissue is highly similar to that of humans, and it is easy to realize whole-body optical visualization and high-throughput screening; zebrafish stands out among many animal models of mental disorders. Here, valuable qualified zebrafish mental disorders models could be established through behavioral test and sociological analysis, which are simulated to humans, and combined with molecular analyses and other detection methods. This review focuses on the advances in the zebrafish model to simulate the human mental disorders; summarizes the various behavioral characterization means, the use of equipment, and operation principle; sums up the various mental disorder zebrafish model modeling methods; puts forward the current challenges and future development trend, which is to contribute the theoretical supports for the exploration of the mechanisms and treatment strategies of mental disorders.

## 1. Introduction

Mental disorders refer to a collection of clinical conditions that stem from brain dysfunction and result in various challenges, including emotional, cognitive, behavioral, and consciousness issues. Research on mental illness has become one of the most important research topics with clinical relevance. However, the etiology of mental disorders is complex, and patients often exhibit symptoms of comorbidity [1].

Mental disorders can be classified into eight categories based on the “Diagnostic and Statistical Manual of Mental Disorders V (DSM-5)” [2]. Eight categories of zebrafish mental disorders based on the DSM-5 have been developed including (a) central nervous system (CNS) disease, including Alzheimer's disease (AD), Parkinson's disease (PD), posttraumatic stress disorder (PTSD), and epilepsy; (b) mental disorders caused by psychoactive substances or nonaddictive substances: psychoactive substances include stimulants, opioids, and marijuana, while nonaddictive substances include toxic mental disorder or withdrawal syndrome; (c) affective disorders characterized by continuous high or low mood, such as depression; (d) personality disorders, for example, autism spectrum disorder (ASD); (e) schizophrenia; (f) Huntington's disease (HD); (g) conduct disorders, such as attention deficit hyperactivity disorder (ADHD); (h) adolescent mental development disorders and other disorders include amyotrophic lateral sclerosis (ALS). The classification of mental disorders is of great significance. Firstly, clinical research in psychiatry requires a globally consistent standard for the diagnosis and classification of mental disorders so that clinical research data can be comparable. Secondly, the clinical diagnosis and classification criteria of mental disorders are very important for the treatment. Clinical treatment depends largely on clinical diagnosis. Thirdly, there is no unified classification and diagnostic criteria for disease prevention and rehabilitation, because different diseases have different prevention and rehabilitation treatments. Therefore, starting from basic medical research, it is necessary to adopt targeted research programs for specific diseases.

**Figure 1:**
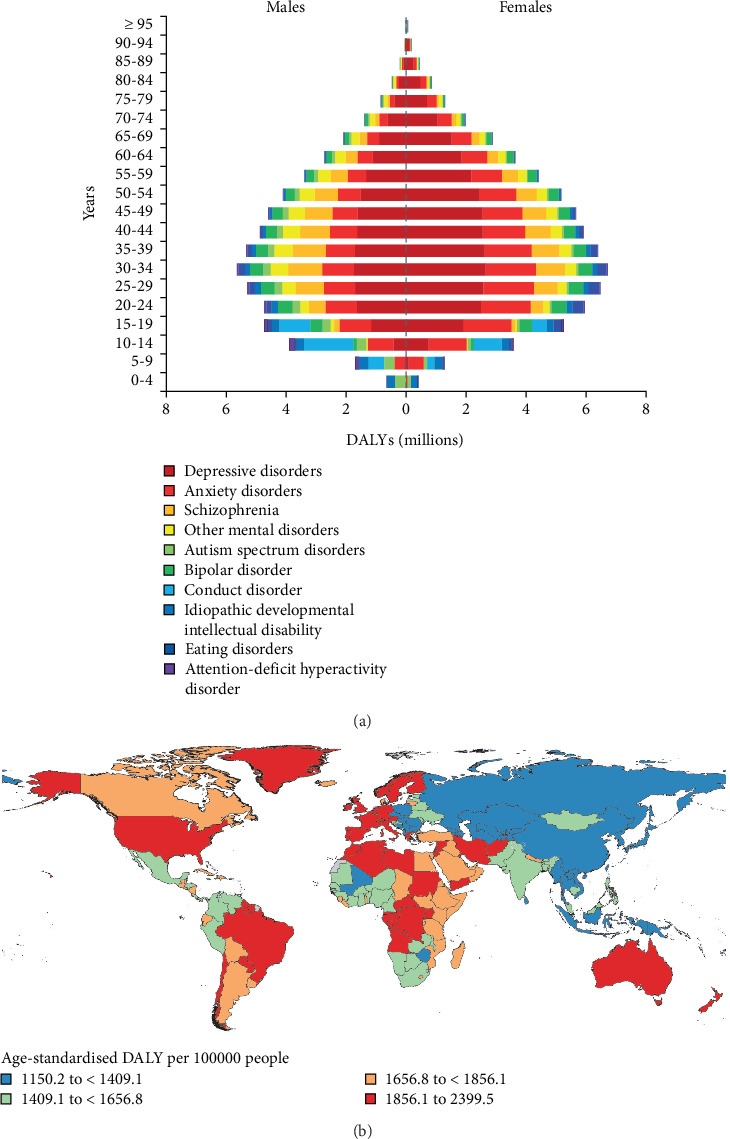
Prevalence and burden estimates of mental disorders, 1990 to 2019, for 204 countries, to measure the global, regional, and national prevalence of mental disorders, disability-adjusted life years (DALYs) [6]. (a) Global DALYs by mental disorder, sex, and age, 2019. Mental disorders ranked from highest to lowest based on total age-standardized ratios in 2019 were anxiety disorders, depression, other mental disorders, idiopathic developmental intellectual disability, attention deficit syndrome, conduct disorder, bipolar disorder, ASD, schizophrenia, and eating disorders. Among mental disorders, depression ranks highest among all age groups except the 0-14 age group, where conduct disorders are the leading cause of burden. The ranking of mental disorders varies by gender and age. (b) Age-standardized DALYs per 100 000 attributable to mental disorders, 2019. The global distribution of DALY for mental disorders by country in 2019 was similar to the trend of the prevalence of mental disorders. The highest DALY rates are found in the United States, Australia, New Zealand, Brazil, parts of Western Europe (e.g., Greenland, Portugal, Greece, Ireland, Spain), sub-Saharan Africa (e.g., Uganda), and North Africa and the Middle East (e.g., Palestine, Lebanon, Iran). The lowest DALY rates were found in Southeast Asia (e.g., Vietnam, Myanmar, Indonesia), East Asia, China, North Korea, high-income Asia Pacific (e.g., Brunei), and Central Asia (e.g., Azerbaijan). Reproduced with permission from [6].

According to World Health Organization (WHO), mental disorders are "the most serious health problem" in the 21st century (Figure 1). WHO projected that by the year 2023, depression would be the second most significant global burden after cancer. People with severe mental disorders have a higher risk of dying up to 20 years earlier than the general population due to preventable physical conditions. There are still significant barriers to mental illness treatment, and coverage of effective treatments with high economic impact remains extremely low [3]. There is an urgent need to identify and expand the availability of drug treatment strategies [4]. In recent years, significant efforts have been made in the field of biopharmaceutical research and drug development for mental disorders, to study etiology, pathology, molecular mechanisms, and drug therapy using rodent models as the primary research objects [5]. However, a comprehensive understanding of the etiology, pathogenesis, and optimal treatment of mental disorders is lacking due to several limitations on the use of rodents, including high economic costs, which may affect sample size and ultimate statistical efficacy. It is essential to expand the animal model toolbox and develop a suitable and effective biological simulation model of humans to investigate the mechanisms that underlie mental disorders. These measures can provide alternative strategies for studying mental disorders and developing targeted antipsychotic drugs.

Therefore, to increase the accuracy of model establishment, it is necessary to identify a suitable model organism. Zebrafish (*Danio rerio*), fruit flies, roundworms, and other earlier "replacement" model organisms are also evolving [7]. Recently, Chinese scientists confirmed that humans evolved from fish. The journal Nature reports on the origin and earliest evolution of jawed vertebrates, which provide key evidence for the transition from fish to man [8–11]. This suggests that fish would have great credibility as a model animal for research. Past research has confirmed that about 82% of human disease genes have homologous genes in zebrafish, with 69% lineal homologous genes [12]. Moreover, the CNS of zebrafish is arranged similarly to that of other vertebrates, and numerous proteins associated with mental disorders in humans are homologous and easily detected [13]. In recent years, neurology research has therefore made tremendous strides, a large contribution comes from the zebrafish which are superior to rodents for the replication of models of mental disorders [14, 15]. Moreover, the emergence of transcriptional activator-like effector nucleases (TALENS) and CRISPR-Cas has significantly changed zebrafish research. Over the past decade, clustered regularly interspaced short palindromic repeat sequences/CRISPR-associated protein (CRISPR-Cas) has enabled the loss of function or acquisition of rapid mutations in target genes, and the potential impact on drug development is undoubtedly enormous. Therefore, with the discovery of multiple genes associated with human diseases, strategies that should be developed to translate these theories into effective treatments have become increasingly important. Zebrafish may be a popular option for investigating candidate mental disorders genes, compared with rodents, due to their simple mutation regime and prominent phenotype.

Given the unclear etiology and poor understanding of the molecular mechanisms of mental disorders, the utilization of zebrafish models is poised to enhance our comprehension of the mechanisms and provide a platform for drug screening. Hence, this review explores recent research findings on the zebrafish mental disorder model, summarizes the modeling methods for mental illness zebrafish models, with particular emphasis on the various behavioral testing methods that are the core index as the model validation strategy, and presents the current challenges and future research perspective.

## 2. Advantages of Zebrafish as a Model of Mental Illness

Zebrafish is a kind of tropical freshwater fish that inhabits India and other countries. It is small in size, develops quickly, and generally attains sexual maturity at the age of 3 months [16]. Adult zebrafish can lay 300 to 500 eggs at a time, and the diameter of the embryos is 0.5~1 mm [17]. Within 3 days of fertilization, embryos are transparent and easily observable to the naked eye. On the third day of incubation, embryos become juvenile.

Generally, zebrafish are widely used in modern drug screening and toxicological analysis due to their large sample size and high economic benefits [18, 19]. Moreover, zebrafish show similar behaviors as rodents compared with other small animals, such as worms and fruit flies [20, 21]. The use of zebrafish in the study of neurodegenerative diseases and neuropharmacology has increased owing to its various advantages such as smaller size, lower costs, well-sequenced genomes, and precise anatomical descriptions. Studies have identified a lineal homolog of genes associated with mental disorders in zebrafish has also been confirmed [22]. Modern molecular biology has enabled the construction of transgenic zebrafish models using techniques such as gene-editing technology [23]. Zebrafish has similar sociological behaviors to those of humans [24]. To establish mental disorder models, it is necessary to develop an animal behavior analysis system to track and analyze the abnormal behavioral symptoms of zebrafish. This approach should be complemented by molecular-level verification to ensure accuracy and effectiveness.

Importantly, there is growing evidence that several key brain regions in zebrafish function similarly to those in humans [25]. On the one hand, in zebrafish, the telencephalic regions of the lateral cortex associated with memory, the hippocampus, and the amygdala cause startle responses [26]. Especially, the dopaminergic system of zebrafish is located in the substantia nigra region of the ventral midbrain similar to that of humans [27]. On the other hand, the brain regions of zebrafish exhibit a remarkable capacity for regeneration [28]. This shows that, compared to traditional mammals, zebrafish can be effectively used to study neural repair processes, hence improving experimental efficiency. In addition, the blood-brain barrier (BBB) was not fully developed before 10 dpf (days postfertilization) in zebrafish [29, 30]. Therefore, this property can be used to achieve drug administration by exposing the embryo or young fish to drugs that may have antipsychotic effects in other model organisms that have not been shown to have therapeutic effects.

Currently, the application of zebrafish *in vivo* brain imaging technology has improved our understanding of the brain function of zebrafish, allowing real-time recording of neuronal activity in real-time. Even though functional magnetic resonance imaging (fMRI) can be utilized to observe the entire brain, it does not provide high resolution at the single-cell level. *In vivo* multichannel and calcium imaging techniques can provide good single-cell resolution but this is limited to a specific brain region. The brain length of juvenile and adult zebrafish is about 1.5 mm and 4.5 mm, and the number of neurons ranges between 10 [5] and 10 [7] [31–33]. Interestingly, zebrafish models allow simultaneous single-cell accuracy, and whole-brain recording may be simultaneously achieved in zebrafish [34]. Researchers imaged the entire brain of a free-swimming zebrafish, at a magnification level that allowed them to see individual neurons. With neurons working together and neural activity spreading like ripples across the brain, the resulting image is stunning. Moreover, this is due to the two characteristics of young zebrafish (about 6 days old). On the one hand, the body is transparent, which facilitates whole-brain imaging; on the other hand, the brain is small, and when combined with calcium imaging by light film, the whole-brain imaging speed may be increased (3 Hz).

**Figure 2:**
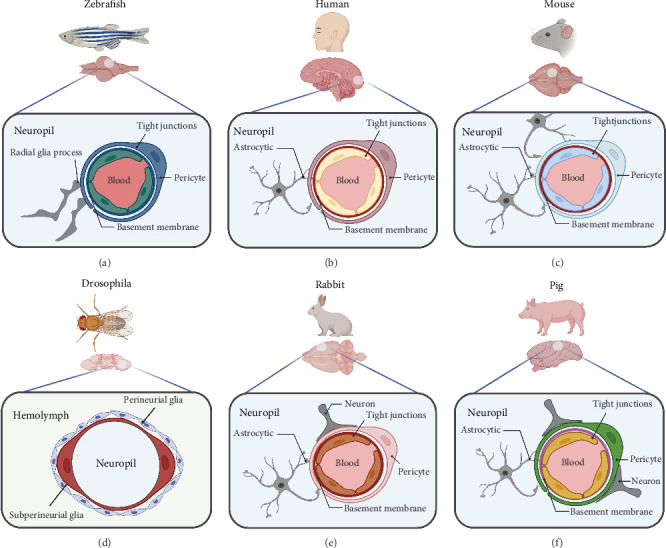
Summary of current BBB models. (a) Similar to mammals, the site of the zebrafish BBB is in endothelial cells, which are connected by the tight junction and are in close contact with pericytes. Zebrafish radial glia process resembles mammalian astrocytes. (b) As in zebrafish, the human BBB site is capillary endothelial cells connected by special tight junctions. Human neurovascular unit also includes pericytes and astrocyte end feet, which are more prevalent in the human brain than in the mouse brain. (c) Mice BBB is also the site in endothelial cells connected by special tight junctions. (d) Hemolymph–brain barrier separates the neuropil from the hemolymph in the fruit fly, hemolymph–brain barrier site in subperineurial glial cells.(e, f) Like humans, rabbit and pig have almost the same BBB structure.

The auditory nerve plays an important role in the establishment of zebrafish as a model animal for mental disorders. Like mammals, zebrafish use hair cells to detect sound vibrations, they lack structures related to the outer ear and middle ear. The hair cells are exposed, and they have a Weberian ossicle chain between the inner ear and the swim bladder. After 5 dpf, the zebrafish swim bladder begins to inflate, allowing sound to resonate in the air before being transmitted to the inner hair cells via the Weberian ossicle chain to produce hearing [35]. In addition, the zebrafish BBB is structurally and functionally similar to that of mammals (Figure 2). At the level of genetic, zebrafish have two homologs of human *abcb1*, namely, *abcb4* and *abcb5*. These were previously designated as *abcb1b* and *abcb1a*, respectively, but were later renamed based on chromosomal localization [36]. The zebrafish *abcb4* protein shares 63% amino acid similarity with human *p-gp*, while *abcb5* shares 57% similarity [29]. Moreover, the immature BBB of zebrafish can generate tight junction proteins *claudin-5* and *ZO-1* at 3 dpf, and the active transport protein *abcb1* can be generated at 8 dpf [29, 30]. It is interesting to note that, similar to mammals, zebrafish can exhibit stress responses, which can be evaluated by animal behavior and other physiological indicators, including heart rate, adrenaline, cortisol, dopamine (DA), and 5-hydroxytryptamine (5-HT), etc. [37]. In addition to behavioral stress responses, zebrafish have significant drug responses, both *in vivo* and *in vitro* [19].

Finally, the clinical symptoms of mental disorders are abstract, and it is currently difficult to quantify and validate animal models of mental disorders using genuine materials. To evaluate the validity of mental models, we still require a significant number of biological samples [38, 39]. The construction of the mental disorders model at the gene level is time-consuming and expensive for mammals. Zebrafish have the advantages of rapid development, large sample size, low cost as well as the fact that symptoms might be manifested in the juvenile stage, making the zebrafish model the dominant organism for the mental disorder [40]. In conclusion, zebrafish is a promising model of mental illness that continues to play an important positive role in the etiology of mental illness and the corresponding medicine screen. The homology of key brain regions between zebrafish and mammals highlights the use of zebrafish models for neurobehavioral and neuropsychological research. In addition, the conservation of neural pathways between zebrafish and mammals allows bidirectional translation of findings. Current genetic tools, tracking techniques, and statistical algorithms are helping to deepen understanding of molecular pathways, develop new compounds or repurpose existing drugs. Combined with the high sensitivity of zebrafish to known antianxiety drugs, antipsychotics, and other psychiatric drugs, this provides researchers with a systematically comprehensive animal model capable of identifying molecular targets for drug therapy and empirically testing their hypotheses.

## 3. Behavioral Characterization of Zebrafish Models in Mental Disorders

Here, in addition to the ecological environment and toxicology studies, the zebrafish model has been increasingly perfected for behavioral analytic techniques in psychiatry [41]. It has gradually become one of the commonly used animal models of mental disorder [42]. he diversity of their behaviors contributed to the diversity of the required behavioral analysis methods, whose primary functions include evoking and measuring specific behaviors [43], observing spontaneous expression behaviors [44], target specific phenotypes and get a range of behaviors [45]. Based on Table 1, zebrafish can perform a variety of behavioral tests, ranging from tests of basic motor and sensory functions to analyses of more complex behaviors related to cognition and emotion.

In the past decade, the field of behavioral research related to mental disorders has undergone a new revolution, and these behavioral characteristics correspond to the extensive and complex network of behavioral phenotypes in laboratory animals. In recent year, the number of behavioral assessments of mental disorders that correspond to model organisms is rapidly increasing to precisely identify the behavioral characteristics of each species that correspond to different disease impairments [7]. To avoid the complexity of experimental design and the selection process and purpose of appropriate tasks, we classified and summarized the existing behavioral analysis methods related to zebrafish mental disorders (Table 1), to aid relevant scientific research in distinguishing and designing behavioral methods corresponding to each mental disorder.

### 3.1. Behavioral of Basic Motor and Sensory Functions

The motor and sensory abilities program aims to assess the normal movement and sensation of the animal. They are critical in animal models of ataxia and asymmetric brain function and are important indicators of recovery after drug therapy.

The motor function tests include a swimming test [46], which refers to the behavioral state of passive swimming of zebrafish under the condition of applying water pressure or stimulation and can measure swimming time and swimming speed and assess individual physical differences. Here, zebrafish have swimming behavior at 12 cm/s water velocity [43]. By establishing a fatigue point, the time at which a fish reaches the fatigue point during a swimming test can be defined. Moreover, the swimming test can determine the degree of fatigue symptoms caused by CNS diseases accompanied by decreased limb motor function.

The sensory function can be evaluated using simple tests. Optokinetic response (OKR) [47] is a classic behavioral test for detecting visual gene mutations in zebrafish. On day 3 dpf, zebrafish will begin to gaze at the moving grating after a period of light adaptation. Furthermore, the startle response, typically an automatic reaction to intense auditory stimuli, can identify any abnormalities in the zebrafish's auditory system. The ability of zebrafish to elicit a fear response and their behavior under this stimulus can be utilized to define behavioral defects in stress response caused by mental disorders. Normal zebrafish react quickly to startle and turn away from the source, such as a device designed by Eddins et al. [48], while Sean et al. [49] stimulated multiple fish simultaneously by tapping. Meanwhile, visual conditioned stimulus and the unconditioned stimulus consisting of an electric shock across the arena [50] are applied throughout the development of zebrafish from juvenile to adult, and the test of zebrafish's preference for fear stimulation can also be realized by using strategically visual cues paired with electroshocks. These tests are similar to the Pavlovian fear conditioning and eyeblink conditioning in mammalian behavior tests. It is suggested that zebrafish model is a rapid, reliable, sensitive, and easy-to-establish animal model.

### 3.2. Depression-/Anxiety-Like Behavior

Mental disorders, such as CNS disorders, psychoactive substances, or non-addictive substance disorders are characterized by anxiety-like or depression-like behaviors. Although these two states cannot be directly measured, these behavioral tests can quantify specific behaviors that change as a result of anxiety/depression. Notably, zebrafish are predominantly active during the day [51]. Since stress is directly linked to depression and anxiety, the hypothalamic-pituitary-adrenal (HPA) axis secretes cortisol, a stress hormone, and reflects the degree of the stress response. As the HPA axis is regulated by circadian rhythms, all anxiety/depression-related behavioral tests are best performed in the afternoon (5-8 h following light exposure) [52].

Anxiety-/depression-like behaviors are mainly studied in combination with animals' exploration behaviors in different environments. This could explain the widespread usage of the Novel tank test (NTT) in anxiety/depression-related studies. Importantly, a 2018 report showed that both anxiety and depression are highly susceptible to comorbid disorders [53], which nearly demonstrates the coexistence of the two states. For example, zebrafish with anxiety and depression exhibit a frozen behavior and hypoactivity phenomenon in NTT. Unexpectedly, extended usage of antidepressant medication induces anxiety, necessitating a division of medication for depression and anxiety to prevent overmedication. Importantly, three factors need to be considered when analyzing anxiety-/depression-like behaviors: first, automated behavior analysis systems are needed due to the large amount of data generated by the task. Second, open-air experiments tend to produce fish adaptations; hence, it is recommended that each animal be exposed to the task only once. Third, the age of the animals, their familiarity with the new environment, and the lighting conditions may influence the results of anxiety-/depression-like behavioral experiments.

Behavioral analysis methods in this category mainly include NTT, shoaling test, light/dark tank test, acoustic startle response, video-tracking experiment, and novel object approach test.

NTT is often used to characterize anxiety-/depression-like levels in zebrafish and can be used to explain the reduced activity of zebrafish under anxiety/depression conditions [54]. In NTT, the position of the fish's center of gravity was determined, and the analysis of the total swimming distance and the time spent on the top of the fish tank should begin 15 minutes after the fish are transferred into the tank. Moreover, anxiety is predominantly demonstrated through erratic movements, which entail a surge in the duration of freezing and swimming toward the bottom. Depression is characterized by reduced irregular movement associated with reduced swimming distance and speed due to developmental delay [55]. Although the NTT has limited representations in distinguishing anxiety- and depression-like behaviors and requires the biochemical level to accurately infer, the shoaling test effectively compensates for this deficiency [56]. The shoaling test is mainly analyzed based on the distance between fish, and the increase in distance between fish indicates depression.

In the light-dark tank test, anxious and depressed zebrafish induced by PD were more likely to swim to the dark tank, which was explained by the innate behavioral trait of zebrafish to avoid the light environment [57]. It is a natural tendency to prefer protection in the dark, comparable to a rodent behavioral trait [58].

The acoustic startle response is used to characterize the phenomenon of reduced stress response speed due to motor retardation caused by depression from sensory function analysis, such as slow response, low movement speed, and decreased distance when avoiding the startle sound source. In addition, acoustic shock may also be used to reflect a decrease in the ability to respond negatively to symptoms of schizophrenia [59]. This method is primarily used to characterize the severity of symptoms by stimulating zebrafish with different decibels levels and digitally analyzing the body angle of zebrafish after being frightened.

In zebrafish, anxiety is characterized by their repetitive behavior. In a previous study, a video-tracking experiment was used performed to examine the anxiety-related behavior among juvenile fish in a 6-well plate. The test parameters indicated that the repetitive behavior of anxious fish was characterized by a decrease in angular velocity and turn angle [45]. In a recent study, a novel object approach test was performed to examine the impact of nicotine on zebrafish boldness. The test involved observing the fish's inclination to investigate unfamiliar objects, which served as a measure of their boldness. Interestingly, this also exposed signs of anxious behavior in the fish [60].

### 3.3. Behavioral of Learning and Memory

Mental disorders such as depression and CNS disorders can cause learning and memory abnormalities, which are associated with brain neuron and nervous system damage. Zebrafish have had a resurgence in learning and memory-related behavior following the discovery of methods to manipulate genetic material and induce targeted mutations. The list of cognitive tests in this field is extensive and diverse. Sometimes, behavior tests require stimulating the appetite motivation of the zebrafish to seek a reward to promote their entry into the target area, and sometimes, they require aversive stimuli that can cause pain or discomfort and promote avoidance or discourage their attempt to enter the target area.

However, all of these studies look at different neurobiological mechanisms. Based on the fact that different nervous systems constitute memory processes, the cognitive tasks are designed to focus on the nervous system. At least five different nervous systems are involved in the memory process: the hippocampus, amygdala, dorsal striatum, nasal cortex, cerebellum, and prefrontal cortex.

Common zebrafish learning and spatial memory tests include the spatial learning test [61, 62], *T*-plus test [63], *Y*-plus maze [64], and plus maze [65]. The principle behind these methods is to determine the characteristics of learning and memory performance by determining the time spent in the target arm or target area. For example, Shovit et al. [61] confirmed through behavioral analysis that high blood glucose can lead to a decline in the learning and cognitive ability of adult zebrafish, and studies have shown that diabetes can lead to depression and other complications [66]. In this test, the *T*-plus maze test was performed to demonstrate the feasibility of zebrafish as a depression-related model. Recording the freezing bouts and freezing duration (s) of zebrafish revealed that zebrafish with mental disorders had more freezing bouts and longer freezing duration (s). The primary parameters used to measure detection in the experimental equipment are the frequency of accessing the target arm or region and the total number of arm visits.

In addition, these learning and spatial memory assessment methods can be combined with color cards for dual-condition assessment. It has been demonstrated that zebrafish can recognize color, and different colors on each arm map are used to determine whether depressed zebrafish have cognitive impairment to color based on the plus maze and *T*-plus test. This also contributes to the development of spatial memory methods combining feeding with two-condition color design [67].

In avoidance conditioning, zebrafish can learn to associate visual cues with potentially harmful stimuli. After contact, vision triggers avoidance behavior, suggesting avoidance of harmful stimuli, or it can trigger a freezing response, which is indicative of elevated anxiety levels [55, 68].

### 3.4. Social Behavior

The social behavior test examines the preferences of zebrafish toward new social goals. This category includes parental, sexual and reproductive, and aggressive behavior in rodents. There are a variety of tests in this category, ranging from those that observe and study social interactions in laboratory animals to those that investigate the formation of social hierarchies in zebrafish populations. Behavioral methods that can be applied to the study of psychiatric disorders in zebrafish include the social interaction test, mirror biting test, shoaling test, predator avoidance test, social preference test, cluster analysis, and conditioned place preference (CPP).

Here, the most prevalent social behaviors associated with mental disorders mainly include aggression or social avoidance. For example, negative symptoms of schizophrenia can manifest as aggression, whereas autism can result in social avoidance. The mirror-biting test [43, 59, 69] can be used to represent the aggressive behavior of schizophrenia patients. To observe biting behaviors and measure aggressiveness levels, a mirror is positioned on one side of the test tank. The time taken for the fish to bite the mirror and their proximity to the mirror are recorded as indicators of their level of aggression [69]. Zebrafish will collide with the mirror image. Currently, the design and assembly of the lens may digitally analyze the times of collision to indicate the degree of aggressive behavior.

Zebrafish inherently exhibit shoaling behavior. Under special conditions such as autism, the shoaling behavior decreases as the distance between fish which represents the degree of autism increases [44]. Similar to the shoaling test, the social interaction test [63] demonstrates the inclination of a zebrafish's behavior toward social interaction. In their natural state, zebrafish exhibit avoidance-like behavior toward predators. Zebrafish behave differently when staying with mates and predators. Researchers have combined social interaction tests and predator avoidance tests to analyze zebrafish anxiety levels [69].

The social preference test, which utilizes a container divided into five compartments, has demonstrated that fish have a preference for social novelty. The frequency with which the subjects are exposed to the unfamiliar fish is examined [70]. Cluster analysis presents unexpected applications for evaluating zebrafish social behavior. Cluster analysis has been developed for measuring overall group cohesion in laying hens [71].

CPP is commonly used to investigate the effects of neuroactive substances on zebrafish. As in rodents, CPP is believed to reflect the motivational properties of drugs and their potential for abuse [72, 73]. Zebrafish typically demonstrate a psychological preference for environments containing particular psychoactive drugs, indicating the value of the CPP model of drug reward in zebrafish [74, 75].

In summary, the zebrafish model offers a unique opportunity to study complex behaviors in a genetically tractable organism, providing valuable insights into the mechanisms underlying human mental processes and disorders. Moreover, the choice between using larval or adult zebrafish in behavioral experiments depends on several factors, including the specific aims of the study, the complexity of the behaviors being investigated, and especially the practical aspects of working with different developmental stages. In the behavioral analysis of basic motor, larval zebrafish display a limited repertoire of simple behaviors, such as swimming and escape responses, while adult zebrafish exhibit a much wider range of complex behaviors, including social interactions, learning, and memory. Larval zebrafish are often used in studies focusing on early developmental processes, such as neurogenesis, neural circuit formation, and the effects of genetic mutations or environmental factors on embryonic and larval development. Importantly, zebrafish develop to 96 hpf (hour postfertilization) the detection of fear responses becomes more challenging due to the increased mobility of juvenile zebrafish. Consequently, around 72 hpf is considered the optimal time for studying zebrafish visual-motor behavior, such as OKR [47], and visual conditioned/unconditioned stimulus [50], while acoustic startle response [59] and video-tracking experiment [45] can be considered after 72 hpf. Larval zebrafish are small, transparent, and relatively easy to handle in large numbers, making them suitable for high-throughput screens and rapid assessments of behavioral responses to genetic or pharmacological manipulations. Behavioral analysis of learning and memory is typically conducted using adult zebrafish models, as this is related to the development of a mature brain system. For instance, in the case of some age-related diseases, adult zebrafish are more suitable for simulating human disease models. Moreover, adult zebrafish are more comparable to humans in terms of their social behavior, as both species demonstrate intricate social interactions and hierarchies.

## 4. Zebrafish Model in Mental Disorders

### 4.1. CNS Diseases

CNS diseases are neurosis, which is one of the major diseases plaguing human health, including anxiety disorder, and CNS syndrome with sensory and consciousness disorders [76]. Its main characteristics include recurrent disease, unclear pathogenesis, and treatment difficulty [77]. Because they do not regenerate or repair easily, nerve injuries may cause several CNS disorders and effective treatments are lacking. Neurosis includes PTSD, AD, PD, and epilepsy [78–81]. The development of new CNS drugs is slow, expensive, and fraught with failure. These challenges stem from the fact that only a few targets for CNS disorders are known and because establishing faithful animal models for CNS diseases is difficult. For instance, psychiatric manifestations, such as depression and psychosis, are difficult to simulate in animals and neurodegenerative endpoints are often difficult to quantify.

**Figure 3:**
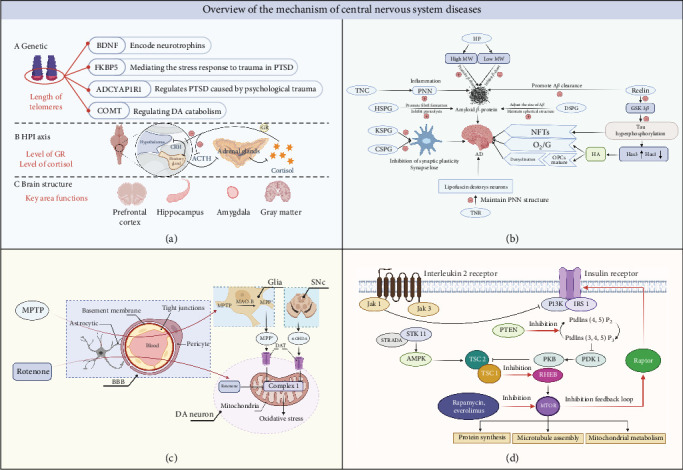
Overview of the mechanism of CNS diseases. (a) PTSD. (A) Shorter telomeres increase the risk of stress-related illnesses and early death such as PTSD in susceptible people. Traumatic events in childhood have also been linked to shorter telomere lengths in PTSD patients. BDNF encodes neurotrophin that plays an important role in the growth, differentiation, maturation, and survival of immature neurons as well as in the regulation of prominent plasticity, neurotransmission, and receptor sensitivity in mature neurons. Molecular chaperone FK506-binding protein 5 (FKBP5) is a key regulator of the stress hormone system that mediates the response to traumatic stress. The association of FKBP5 gene polymorphism with childhood maltreatment can predict the severity of PTSD symptoms [82]. ADCYAP1R1 is a high affinity receptor for PACAP, which is related to estrogen-dependent regulation. Genetic susceptibility to PTSD may depend on PAC1 expression in the cortex [83]. COMT (Catechol-O-methyltransferase) regulates cortical function, and there is a gene-environment interaction between the Val158Met polymorphism in human COMT and the type of traumatic events experienced in the risk of PTSD [84]. (B) The level of GR and cortisol in the HPI axis. Glucocorticoids are mediators of the HPA axis, mediated by GR, and then exert effects on physiological functions. Lower cortisol levels in morning specifically correlate HPA axis dysfunction with PTSD hyperalert symptoms [85]. (C) The key brain regions associated with PTSD symptoms were the prefrontal cortex, anterior cingulated cortex, amygdala, and hippocampus, and they are involved in the formation of emotion and fear memory and recovery. (b) Extracellular matrix (ECM) levels of AD mechanisms. One of the manifestations of AD is the increase of TNC (Tenascin-C), which induces inflammation. TNC promotes the stability of PNNs (perineuronal net) and reduces the scavenging capacity of A*β* protein. HP (heparin) and HSPGs (heparan sulfate proteoglycans), DSPGs (dermatan sulfate proteoglycans) and HA (hyaluronic acid), and TENASCIN-R (TNR) are increased in AD. HP with different molecular weights (MW) affects the generation of A*β* by regulating the *β*-sheet secondary structure. HSPGs promote the formation of A*β* fibrils, inhibit amyloid proteolysis, and promote A*β* production. DSPGs maintain the stability of A*β*. HA inhibits the maturation of OPCs, causes demyelination, and reduces the supply of brain oxygen and glucose (O2/G). TNR may prevent lipofuscin from destroying neurons. Others like reelin and keratan are decreased in AD. Reproduced with permission from [86]. (c) Pathways and injury mechanisms of dopaminergic neurotoxins in PD. Here, 6-OHDA (6-hydroxydopamine) reaches dopaminergic neurons via the SNc (substantia nigra pars compacta) or the striatum. MPTP (1-methyl-4-phenyl-1,2,3,6-tetrahydropyridine), rotenone, and paraquat can cross the BBB. In this study, the metabolite of MPTP, MPP+, is transported to dopaminergic neurons via DAT (dopamine transporter). These substances induce oxidative stress, ultimately leading to the neuronal death. Reproduced with permission from [87]. (d) Mechanism of epilepsy mediated by mTOR signaling pathway. Reproduced with permission from [88].

The use of zebrafish to verify behavioral models has potential application in CNS drug discovery, which benefits from their behavioral phenotypes, genetic factors, and pharmacological sensitivities are generally similar to those reported in rodent brain disease models and human clinical populations. Besides, imaging the activity of the CNS is an important way to identify the state of the brain. The small size and optical transparency of zebrafish larvae make it possible to image *in vivo* at high resolution and manipulate neural activity in living animals. By modulating these proteins and cells, antipsychotic drugs mediate specific and unique behavioral changes that can uncover suitable compounds. In zebrafish behavioral tests, some known neuroactive drugs produce unique and reproducible behavioral effects and the development of corresponding high throughput behavioral models can identify new compounds with similar behavioral effects. Alternatively, genetic engineering can be used to introduce human disease mutations in zebrafish. After mutation, compounds that counter abnormal behavior and restore normal behavior can be identified. Taken together, disease models based on zebrafish have the potential to uncover molecular level strategist for treating CNS diseases (Figure 3) (Table 2).

#### 4.1.1. PTSD

PTSD is a mental disorder caused by sudden mental stimulation, such as personal experiences of traumatic events like war and major accidents. Its clinical manifestations include excessive vigilance, insomnia, and dreams that are accompanied by flashbacks of past traumatic events [89]. PTSD was first identified in soldiers returning from war and PTSD patients experience long-term recurrent nightmares and sleep disturbance, which in severe cases, may cause depression and other mental disorders [90]. Studies have shown that PTSD can lead to varying degrees of damage to the prefrontal cortex [81]. Because the zebrafish brain is highly similar to the human brain, zebrafish can model human PTSD.

Like humans, zebrafish release cortisol, a biochemical indicator of stress intensity, in response to environmental changes or mental stress [91]. Moreover, zebrafish allows convincing comparison with humans because it exhibits a wide variety of behaviors, including complex learning and neurobiological changes. Zebrafish also exhibit individual differences (extroversion, biological makeup, and sex differences) [92, 93]. Several methods, mainly based on giving zebrafish stressor stimulation tests, can be used to quantify stress responses and associated response factors in zebrafish. Furthermore, several clinical biomarkers for PTSD, including glucocorticoid receptor [94, 95], adrenocorticotropic hormone [96, 97], neuropeptide Y [98, 99], catechol O-methyltransferase [100] and monoamine oxidase A/B have homologs in zebrafish and have been used to develop a PTSD-like model [101].

The pituitary adenylate cyclase-activating polypeptide receptor (PAC1, also known as ADCYAP1R1) is relevant to PTSD and generally, the modulation of stress responses [102, 103]. To examine the specific role of the *PAC1-hop* splice variant, Jakob et al. generated a zebrafish mutant bearing a deletion in the alternatively-spliced hop exon of the *pac1a* gene. COMT is thought to be a heritable predisposing factor for PTSD. Additionally, the human tryptophan hydroxylase, *TPH1* and *TPH2*, are also associated with PTSD risk [104].

Although zebrafish are feasible as PTSD models, their environmental stressors are completely different from those affecting humans. However, simulation of human traumatic stresses like electrical stimulation, stressors that are similar to each link in chronic unpredictable mild stress (CUMS), and simulation of traumatic stress conditions like abuse and forced separation from social groups in the process of reproductive development may still be modeled in zebrafish.

#### 4.1.2. AD

**Figure 4:**
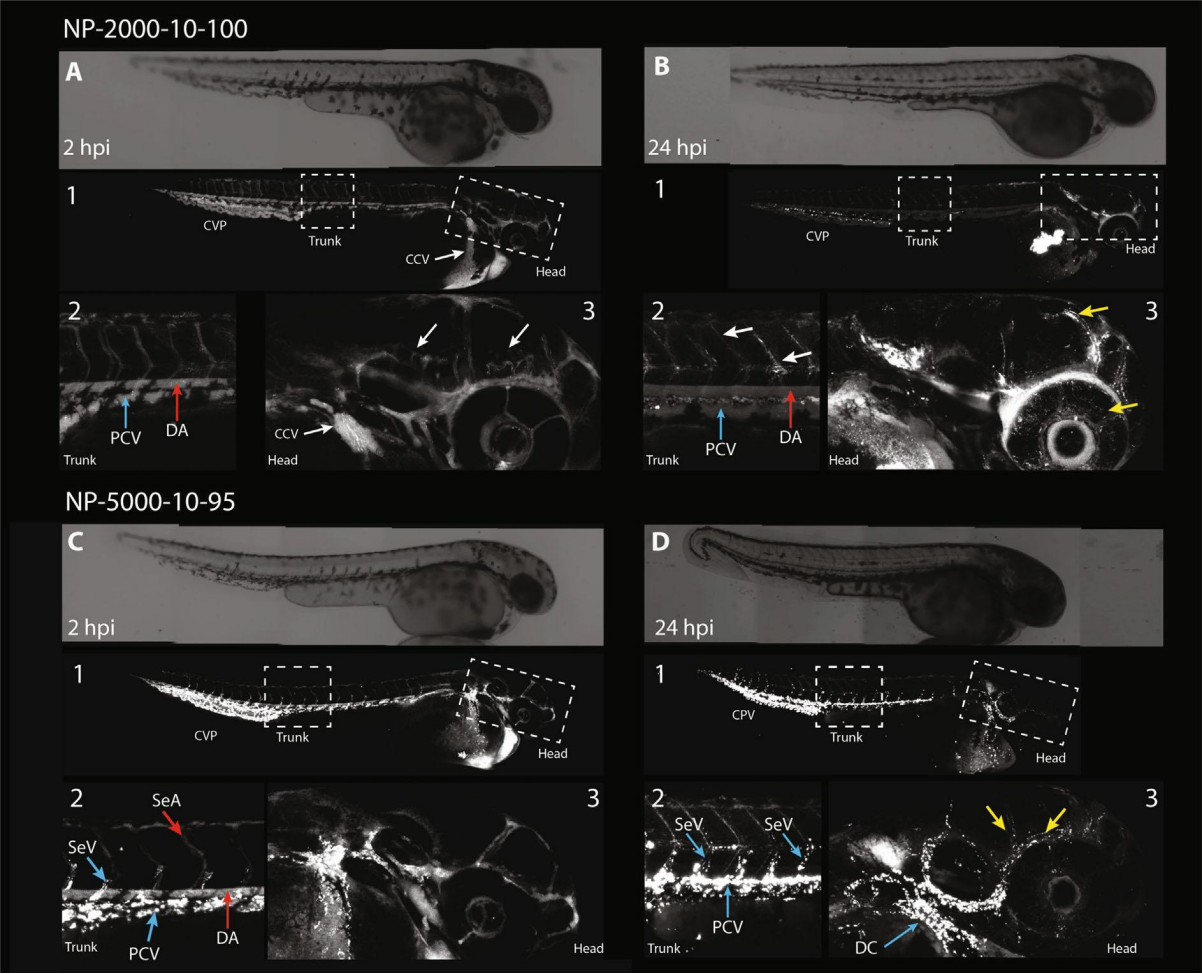
The nanoparticles injected into the zebrafish pass through the blood and are deposited around the cerebral blood vessels. Diblocks of the polymeric material PEG-b-PLA were created through the process of ring opening polymerisation, utilizing either PEG2000 (A, B) or PEG5000 (C, D). After imaging, the caudal vascular plexus (CVP), posterior common vein (CCV), dorsal artery (DA), and posterior cardinal vein (PCV) can be seen clearly. The blue arrow represents venous blood vessels, and the red arrow represents arterial blood vessels. Reproduced with permission from [112].

AD is a progressive neurodegenerative disease that causes cognitive deficits, delusions, hallucinations, and changes in mood and behavior, which was first identified by Dr. Alz during a cranial autopsy on an elderly patient [105]. Clinically, AD is characterized by the secretion of the insoluble, extracellular amyloid-*β* (A*β*) protein into the brain, aggregation of neurofibrillary tangles (NFTs) in the CNS, and neuronal deficits. AD patients experience progressive cognitive decline, such as impaired visuospatial skills, executive dysfunction, and behavioral changes [106, 107]. Because of its biological background, zebrafish has the potential to model AD. For example, the lateral cortical areas of zebrafish are highly homologous to those of human, contributing to build the stable AD models [108, 109]. Current AD models are based on A*β* aggregation [110], with the auxiliary inclusion of some AD-related biomarkers, such as DA and noradrenaline (NA) dopaminergic, as well as the degree of neuronal damage. Ver et al. [111] described how PDE5 inhibitors, which include the erectile dysfunction drugs, sildenafil and tadalafil, decrease the accumulation of mutant proteins and reduce cell death and anatomical defects in zebrafish models of neurodegeneration. A group of disorders is caused by the abnormal accumulation of tau protein, including HD and tauopathies, AD, and frontotemporal dementia [111].  Figure 4 illustrates the use of zebrafish as a model for delivering nanoparticles to the brain to treat neurodegenerative diseases such as AD and PD [112]. It is also reported that okadaic acid (OKA) effectively inhibits the activity of protein phosphatase 2A (PP2A), which causes neuroinflammation. Because PP2A-mediated suppression of the activity of Tau phosphorylase indirectly inhibits the production of NTFs, frequently used as a modeling reagent [80]. Gao et al. treated adult (≥12 months ole) zebrafish with 100 *μ*M OKA [113] and observed significant NTFs clustering in the brain on day 9. A study in which zebrafish embryos were treated with benzo[a]pyrene [114], a pentacyclic polycyclic aromatic hydrocarbon, from 3 dpf, found that benzo[a]pyrene-treated adult zebrafish exhibited motor and cognitive decline, reduced levels of DA, dihydroxyphenylacetic acid, and norepinephrine (NE), as well as dopaminergic neuronal damage. In addition, A*β* aggregation, significant apoptosis of neuronal cells, and longer swimming distance in the lower part in NTT are typical symptoms of AD [114].

Although wild-type zebrafish seem to lack some neuropathological features of AD, the advent of gene editing technology has expanded the use of zebrafish in the study of amyloid and neurogenic fiber entanglement. Keturah et al. [115] detailed how through gene editing, zebrafish can be used to study the causes, pathology, molecular mechanisms, and drug targets of AD. Study has modulated the expression of *APPsw* in zebrafish and assessed the effects of A*β* on cerebrovascular injury and animal behavior [116]. *APPsw* mutations induce APP overexpression, which in turn, promotes A*β* production and triggers AD symptoms. The A*β* amyloid deposition and neuronal damage in zebrafish are similar to that observed in a transgenic mouse model (*Tg2576*) which also has mutant *APPsw*. Moreover, *A152T-Tau* zebrafish exhibited significantly elevated levels of tau protein and motor dysfunction [117]. Metal ions are also widely used to establish animal models of AD [118]. Aluminum (Al) is a neurotoxin [119] that can reduce the activity of acetylcholine and leads to neuronal damage in AD. A study of the therapeutic effects of linarin against AD found that AD symptoms were triggered by continuous exposure of zebrafish embryos to 140 *μ*M AlCl_3_ for 3 days [120]. Copper (Cu) has also been shown to induce AD in zebrafish [121] Moreover, it is reported that vast amounts of Cu^2+^ accumulate in the brain of AD patients and that Cu^2+^ promotes the aggregation of NFTs [122], which may be associated with the roles of the ions in neurotransmission. Other metal ions, including Co [123], Fe^3+^ [124], Zn^2+^ [124], Ag^2+^ [125], Pb^2+^ [126–128], Ni^2+^ [129], Hg^2+^ [130–132], Mn^2+^ [133–135], Cd^2+^ [128, 136, 137], and CrO_4_^2-^ [138] can also be used for AD model construction, which is more cost effective than the use of OKA.

The zebrafish model is highly valuable in exploring the etiology of AD, particularly the potential role of hypoxia as a risk factor. In zebrafish, it is easy to reproduce the hypoxic state by reducing water oxygen levels or by chemical mimicking of sodium azide, allowing mitochondria to release free radicals that increase oxidative stress levels. Similar to humans, hypoxic conditions in larval and adult zebrafish upregulated several genes associated with AD, including *PEN1*, *PSEN2*, *AppA*, *AppB*, and *Bace1* [139–141]. However, whether zebrafish can fully reproduce the molecular pathological mechanism of AD still requires further research data on the structure and physiology of the zebrafish brain, such as the molecular mechanism of *APOE* and *MAPT*.

#### 4.1.3. PD

PD is the second most common neurodegenerative disorder; however, its preventive and therapeutic options are limited [142, 143]. PD is highly prevalent among the elderly, especially those aged over 50 years, with up to 4% of patients being over 85 years [144]. Its clinical manifestations include numbness in the hands and feet, resting tremors, neurological failures, anxiety, and depression. Moreover, due to its wide range of pathological effects, it can lead to some nonmotor symptoms [78]. The main pathological changes in PD involve degeneration of substantia nigra and reduced dopamine levels in brain neurons [145]. Biologically, *α*-syn aggregation and mistransmission underlie the pathogenesis of PD. Mitochondrial dysfunction in PD patients can lead to *α*-syn aggregation [146]. In addition, elevated inflammatory levels have been found in the brains of PD patients [147, 148]. Moreover, human genome-wide association studies have shown that the inheritance of PD is associated with mutations in several genes, including *LRRK2*, *SNCA*, and *PARK7*/*DJ-1* [149, 150]. Developing a model that can accurately replicate the features of human PD, including predicting brain and neuronal damage as well as motor and behavioral deficits, is a difficult challenge. This is due to the fact that these symptoms typically take years to manifest in humans. Compared to other PD animal models, larval behaviors in zebrafish can be evaluated via high-throughput automated methods. In addition, zebrafish is associated with low costs, embryonic and larval transparency, and is easily susceptible to simple genetic modifications, which support easy fluorescent labeling and noninvasive assays, making it an ideal model animal for PD studies [151, 152]. Meanwhile, the regenerative capacity of zebrafish dopamine neurons is much higher than that of humans, and understanding the pathomechanisms involved in neuronal regeneration is important for informing PD treatment. In a previous study assessing the magnitude and variability of the ability of adult zebrafish neuronal cells to regenerate, Caldwell et al. [153] investigated whether the loss of various dopaminergic neuronal populations is sufficient to trigger functional neuronal regeneration. They found that dopaminergic neuronal populations in adult zebrafish brains showed great variations in their regenerative capacity, which was associated with the constitutive nature of neurons. The regenerative function was also dependent on immune system activation. These findings provide new ideas for future treatment of PD presenting with symptoms such as motor abnormalities and tremors [153].

Therapeutic options for PD include pharmacotherapy, gene therapy, cell transplantation, and cell reprogramming [154]. Development of noninvasive, simple, and effective strategies for restoring the functions of endogenous dopamine neurons is associated with various challenges. Piezoelectric composite nanoparticles have been used to modulate neuroplasticity and restore the functions of degenerating dopaminergic neurons *in vivo*. Nanoparticles receiving ultrasound signals can generate electrical signals to be transmitted to nerve cells, stimulating voltage-dependent ion channels in nerve cells and resulting in cell depolarization. Moreover, the nanoparticles increased the expressions of synuclein, a synaptic plasticity marker, and controlled tail movements in zebrafish by regulating the inward flow of calcium ions in neural circuits. DA is a neurotransmitter that regulates the CNS, and its rate-limiting enzyme, tyrosine hydroxylase, catalyzes the conversion of L-tyrosine to L-dopa. The electric field generated by piezoelectric nanoparticles activated tyrosine hydroxylase expressions and improved dyskinesia in Parkinson's zebrafish [155]. Electromagnetic nanoparticles exhibit favorable biosafety characteristics and present novel possibilities for the remote treatment of neurodegenerative diseases and neural regeneration.

Currently, zebrafish PD models are mainly established by using MPTP and 6-OHDA to kill neurons [87]. However, neuronal death mechanisms are not correlated with PD pathophysiology (Figure 3(c)) [87]. Environmental toxins are associated with increased PD risks; thus, zebrafish models have been used to study PD-associated toxins and their pathogenesis. Environmental toxins, including rotenone [156–158], paraquat [159–161], ziram [162], and benomyl [163] increase PD risk by affecting the dopamine nervous system to varying degrees and by producing action effects that can model PD-like behaviors in zebrafish [57, 87, 164, 165]. In addition, zebrafish embryos treated with DEPe (diesel exhaust particulate extracts) also showed a loss of various neurons (including dopamine neurons) and behavioral alterations, leading to PD onset [166]. The severity of PD at the biochemical level is determined through the assessment of oxidative stress. Inflammation and dopamine uptake triggered by ROT and AD caused by oxidative stress are accompanied by a decrease in CAT (catalase) and SOD (superoxide dismutase), reduced GSH (glutathione levels) and GST (glutathione-S-transferase) activity, and other mechanisms including NADPH oxidase activity and increased O^2-^. 6-OHDA is a neurotoxin that inhibits mitochondrial complex I, resulting in mitochondrial impairment [167]. In the meantime, NTT [57, 161], light/dark test [158], touch-evoked escape response, locomotor activity [157–159, 162], object discrimination task [158], preference for conspecifics [161], and aggression test [161] are used to evaluate the Parkinson-like symptoms in zebrafish.

The genes encoding synuclein, *LRRK2*, *GBA*, *Parkin*, *DJ1*, and PTEN-induced *Pink1* (putative kinase1) are several common genetic modification sites used to construct PD zebrafish models. In zebrafish, these genes have a high homology (mostly above 65%) with human genes [157, 168–171]. PD pathogenesis is highly associated with *α*-syn aggregation. Correlations between PD and abnormal mitochondrial functions were revealed in a *Pink1* knockdown model, while *DJ1*^−/−^ zebrafish exhibited elevated risks of PD development by astrocyte overexpression. The highly conserved PD-related genes in zebrafish and humans also include *ATP13A2*, which codes for a lysosomal protein [172]. Mutations in *ATP13A2* promote the induction of Kufor-Rakeb, an early PD that is also known as juvenile PD [173]. A recent study [172] reported that *ATP13A2* knockdown in zebrafish was associated with early motor deficits in zebrafish larvae and exhibited symptoms of juvenile PD. Although complete knockout of the *ATP13A2^−/−^* gene during juvenile development results in embryonic death, further investigation is needed to fully understand the specific mechanisms by which *ATP13A2* is implicated in the development of Parkinson's disease during growth. Besides, Gideon et al. [174] used CRISPR/CAS-9 genome editing to remove the *PARK7*/*DJ-1* gene in zebrafish, resulting in a reduction of dopamine neurons, which in turn significantly reduced the distance, speed, movement, and swimming time of zebrafish. RNA sequencing of zebrafish PD models' brains has unveiled molecular characteristics that closely resemble those of human Parkinson's brains, including elevated levels of oxidative phosphorylation and cell-cycle regulation. In addition, unbiased machine learning algorithms have been used to classify zebrafish dyskinesia which will be widely applicable for assessing zebrafish models of human motor diseases and provide a valuable asset for the therapeutics pipeline. Therefore, we cautiously conclude that the zebrafish PD model is a good way for researchers to investigate the molecular mechanisms underlying PD, how nerve cells adapt to neural circuits, and how potential new therapies might affect these disease processes.

#### 4.1.4. Epilepsy

Epilepsy is a chronic disease that is also referred to as a recurrent, transient brain dysfunction syndrome [175, 176], which is characterized by recurrent spasms/seizures, behavioral disorders, pathological neural activity, and endocrine dysfunction, including congenital epilepsy and psychiatric injury epilepsy [177]. The pathomechanisms involved in epilepsy development are complex. Studies have speculated that it may be caused by the excess firing of pathological brain neurons due to various abnormalities, leading to the synchronous firing of peripheral neurons, followed by epilepsy [178].

Zebrafish epilepsy models are good alternative animal models for studying the mechanisms involved in epilepsy development and for screening antiepileptic drugs (AEDs) [179–186]. Epilepsy can be modeled in larvae and adult zebrafish (primarily through the administration of convulsant and genetic modifications) and assessed by a variety of behavioral and physiological end points. The epileptiform state in adult zebrafish is characterized by behaviors such as hyperactivity, unstable swimming, loss of body posture, spasmodic spiral swimming, and CNS discharge. In addition, biochemical studies involving zebrafish have revealed that hormones, cytokines, and peripheral blood transcriptomes, which are implicated in human and rodent epilepsy, may also be tested in zebrafish epilepsy models [185].

As a CNS convulsant, pentylenetetrazol (PTZ) is commonly used to establish epilepsy model organisms [187]. Mechanistically, PTZ induces epileptic symptoms by interfering with the neurotransmission of GABA, an amino acid neurotransmitter that acts as a sedative for sympathetic excitation. PTZ is a GABA receptor antagonist that promotes the closure of receptor channels [79, 188]. Zebrafish are susceptible to drugs in bathing mediums; thus, drugs can be delivered to freely swimming zebrafish, and locomotory as well as electrophysiology features associated with seizures can be quantitatively assessed [186]. Li et al. used 10 mm PTZ to establish epileptic models of zebrafish (6 dpf) for AEDs screening [189]. In a study conducted in 2019, intraperitoneal infusion of zebrafish with 80 mg/kg PTZ daily for 10 consecutive days was associated with significant seizure symptoms on D4. This study was the first to construct a PTZ-induced kindling model using adult zebrafish [190]; thus, more studies should be performed to confirm the advantages and limitations of model building.

(D, L)-Allylglycine (AG) is an irreversible inhibitor of glutamic acid decarboxylase (GAD), which leads to suppression of GABA synthesis. This process is mediated by the active metabolite of AG [191]. Zebrafish exposed to 50~300 mm AG showed epileptic symptoms at 7 dpf [192]. The AG zebrafish and AG mammalian models exhibited comparable outcomes when supplemented with antiepileptic drugs, demonstrating that AG-induced epilepsy in zebrafish is a valid model.

Red alginate (KA) is a CNS stimulant [193] that mediates the production of excitotoxicity [194]. Previously [195], 50 *μ*M KA resulted in sustained brain neuronal firing in larva zebrafish (5-15 dpf). The experimental group had eight times the firing frequency of the control group. However, 6 mg/kg (i.p) of KA-induced epilepsy in zebrafish (3 to 6 months) showed a 20% mortality rate [196]. These findings imply that glial fibrillary acidic protein (GFAP) may be an important substance that facilitates amino acid transport of KA [197]. Moreover, KA reduced glutamate levels in zebrafish to levels that were similar to those of mammals.

The inhibition of the GABA pathway has been linked to the pathogenesis of epilepsy [198]. Dravet, a genetic epilepsy syndrome in adolescence [199], is attributed to mutations in *scn1a* [198, 200]. *scn1a* in zebrafish and humans have the same functions and characteristics; thus, zebrafish are useful models for studying Dravet. In this study, *scn1a* is directly related to *scn1lab* [201], by labeling and modulating the inhibitory and excitatory ganglia of *scn1lab*. Using CRISPR-CAS9 to verify the similarity of *scn1lab*^−/−^ models with the construction of the Dravet syndrome [40, 202, 203]. Additionally, the EAST/SeSAME syndrome is associated with mutations in *KCNJ10* and *KCNQ3* families [204, 205]. The EAST/SeSAME syndrome is a neonatal epilepsy syndrome. To address concerns related to the off-target effects of gene editing, studies necessitate the use of a conventional approach involving larger sample sizes to ensure the stability of the mutant population [206]. The use of morpholine (MO) to knockdown *got2a* in zebrafish led to microcephaly, pericardial edema, and body curvature. Electrophysiological assessment showed that epileptiform EEG signals and the number, as well as the duration of seizures, were significantly high in mutant zebrafish than in wild type zebrafish. This result confirms the association between *got2a*, the epileptic encephalopathy phenotype, and developmental defects in the brain. Then, the investigators assess the efficacies of therapeutic interventions, using different concentrations of pyridoxine, serine, pyruvate, and proline in zebrafish larvae. The administration of pyridoxine and serine in zebrafish with *got2a* knockdown resulted in the reversal of the severe phenotype, improvement in survival rate, and reduction in the number and duration of seizures. These findings have significant implications for the clinical management of patients with *got2* mutations and associated epileptic encephalopathy [207]. To investigate the role of *ubtor* in epilepsy, the mechanisms of *ubtor* in epilepsy-like behaviors were analyzed via *in situ* hybridization by assessing early embryonic spontaneous movements of zebrafish and the behaviors of drug-induced epileptic zebrafish models [208]. Genes that are relevant for construction of zebrafish epilepsy models include snape protein complexes, such as *stx1b* and *stxbp1* [209], which are responsible for the initiation, docking, and fusion of synaptic vesicles during neurotransmitter release. *cacna1a* [210] encodes the Ca^2+^ channel and its mutations cause Ca^2+^ channel inactivation. Recombinant chromodomain helicase DNA-binding protein (CHD2), which is involved in chromosome formation, indirectly regulates epilepsy development. Hypothetically, *CHD2* is associated with psychiatric complications. More than 70 epilepsy-associated genes have been identified [211]; however, a few genes have not been clinically confirmed, which should be explored further via gene editing. For multiple validations, these genes should be reproduced in zebrafish to establish the underlying mechanisms of epilepsy.

### 4.2. Mental Disorders due to Psychoactive Substances or Addictive Substances

**Figure 5:**
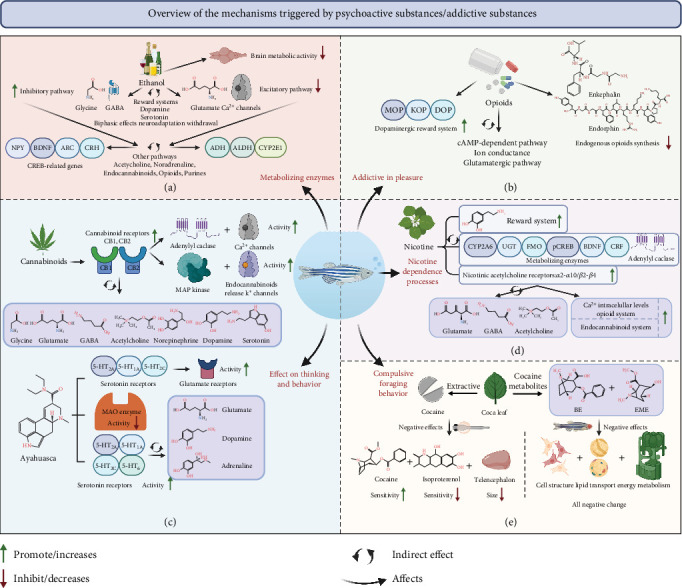
Overview of the mechanisms triggered by psychoactive substances/addictive substances. (a) Based on the effects of ethanol on zebrafish. In zebrafish, it was found that the activity of brain metabolic and glutamatergic neurotransmission was decreased, with the increased of Ca^2+^ permeability and GABA- and glycine-mediated synaptic activity. In addition, ethanol indirectly activated dopaminergic and the activity of serotonergic neuron. Finally, ethanol affects the homeostasis of other pathways, such as cholinergic, noradrenergic, opioid, endocannabinergic, and purinergic systems. The effects of ethanol on zebrafish are complex and interconnected. Ethanol affects metabolizing enzymes such as ADH, ALDH, and CYP2E1; and CREB-related genes, such as NPY, BDNF, ARC, and CRH are modulated by ethanol consumption. (b) Based on the effects of opioids on zebrafish. Opioids positively regulate MOP, KOP, and DOP opioid receptors and activate dopaminergic nervous system, which is also the main cause of addiction. In addition, other pathways such as camp-dependent biochemical pathways, ion conductance, and glutamate pathways are affected. (c) Based on the effects of cannabinoids on zebrafish. Cannabinoids act on CB1 and CB2 receptors to regulate the release of neurotransmitters, including glutamate, GABA, glycine, acetylcholine, NE, DA, 5-HT, and cholecystokinin and then causes the activation of adenylate cyclase, voltage-gated Ca^2+^ channels, and potassium channels, while inhibiting MAP kinase activity and endocannabinoid release. The increase of monoamines promotes addiction. Ayahuasca preparations act on 5-HT1A, 5-HT2A, and 5-HT2C serotonin receptors, and ayahuasca has the property of inhibiting monoamine oxidase, which will lead to an increase in monoamine levels in the brain. (d) Based on the effects of nicotine on zebrafish. Nicotine activates nAChRs (*α*2-*α*10, *β*2-*β*4) and promotes the release of neurotransmitters, including acetylcholine, dopamine (reward system), serotonin, GABA, glutamate, and NE in the brain, then increases intracellular levels of Ca^2+^, and positively modulates the opioidergic and endocannabinoidergic systems. Finally, nicotine directly affects the levels and adenylyl cyclase activity of nicotine-metabolizing enzymes such as CYP2A, UGT, and FMO, as well as pCREB, CRF, and BDNF. (a–d) These are reproduced with permission from [214]. (e) Based on the effects of cocaine on zebrafish. In the zebrafish larvae experiment, cocaine consumption led to an increase in further cocaine susceptibility and a decrease in isoproterenol susceptibility. Tissue samples reveal telencephalon toxicity, characterized smaller size. In adult zebrafish, cocaine metabolite benzoylecgonine (BE) and EME (ecgonine methyl ester) cause negative change in cell structure, lipid transport, and energy metabolism [215].

Generally, the most common mental disorder caused by psychoactive substances is drug addiction. In the guidelines for DSM-5 [2], psychoactive substances that can cause addiction are divided into the following categories [212]: alcohol, opium, cannabis, sedative-hypnotic, and cocaine. Other stimulants include caffeine and hallucinogens and tobacco and volatile solvents. Zebrafish exhibit a diverse range of behaviors that can be thoroughly investigated and described. These behaviors include but are not limited to aggression, anxiety, long-term and short-term memory, object discrimination, and color preference. Indeed, zebrafish can develop mental disorders like humans, including the development of drug resistance and withdrawal syndrome [213]. Similar to humans, zebrafish self-rewarding is dependent on the *μ*-opioid receptor and the two key pathways for drug addiction: the dopamine and glutamate pathways. As shown in Figure 5, zebrafish offer an excellent platform for study into the effects of psychoactive drugs on the CNS and potential treatments for drug addiction based on microbiometric *in vivo* biometrics. Over the past 20 years, zebrafish have been used successfully to assess the physiological effects and molecular mechanisms of psychoactive or addictive substances, encompassing a wide range of complex behaviors, such as aggression, anxiety, long-term and short-term memory, object discrimination, and color preference. Although most of the studies reviewed here used adult zebrafish for behavioral analysis, many behaviors related to psychoactive or addictive substances can also be examined in zebrafish larvae. Finally, zebrafish-based trials will bridge the gap between *in vitro* and preclinical studies in advanced mammalian models, thereby facilitating the discovery of new pharmacological tools and drug clues.

#### 4.2.1. Alcohols

Between 1990 and 2017, the global consumption of alcoholic beverages increased by 70%, from 21 billion to 35.7 billion, while the annual death rate increased to 1.8 million people each year [216]. Although alcohol is addictive, it increases the risk of diabetes, cardiovascular disease, and liver disease and is classified as a level 1 carcinogen by the WHO [3]. Alcohol addiction has serious adverse effects on human health and social stability; however, there is currently a very limited choice of drugs for treating alcohol addiction.

The two main characteristics of alcohol addiction are alcohol tolerance and withdrawal. Movement patterns, physiological characteristics (cortisol levels), and neurochemical changes after acute ethanol exposure and withdrawal can be used to characterize ethanol tolerance and alcohol withdrawal in zebrafish [217]. Several studies have examined the effects of taurine and alcohol on the social and fear responses of zebrafish to determine whether mixing energy drinks with alcohol exacerbates its negative effects [218–220]. These results demonstrate that taurine modulates EtOH-induced anxiolytic- and anxiogenic-like behaviors in a concentration-dependent manner, suggesting a complex interaction mechanism between TAU and EtOH interactions.

In addition to adult alcohol addiction, parental alcohol consumption during pregnancy directly causes lifelong neurological disorders in the fetus, along with a range of cognitive and behavioral disorders [221]. Globally, about eight out of 1,000 children have fetal alcohol spectrum disorders (FASD) [222]. To simulate the effects of moderate prenatal ethanol (MPE) exposure, zebrafish embryos (2-9 dpf) were exposed to 20 mm of ethanol. Adult zebrafish had slight spatial memory, and reduced *Y* maze alternating behavior after 3 months did not affect fish appetite and response to stimuli. This model laid the foundation for subsequent studies on the effects of alcohol on learning [64, 223, 224]. In recent years, studies on the therapeutic effects of various drugs on FASD have taken advantage of zebrafish's faster development when compared with mammals. Severe FASD simulation by exposing embryos to a culture medium containing 100 mm of ethanol 3 h after fertilization revealed that folic acid (FA) inhibits ethanol-induced malformation in juvenile zebrafish [225].

Indeed, there is an urgent need to understand the mechanisms underlying human alcohol addiction and to uncover effective therapeutic strategies. To this end, several rodent models of alcohol addiction have been developed. However, the cost of maintaining animal models is very high. Zebrafish possess a digestive system, including the liver and intestines, that bears striking similarities to that of humans. Consequently, their processes of digestion and nutrient absorption closely resemble those of humans. Excessive alcohol consumption and its metabolism generate high levels of acetaldehyde, which accumulates in the body, causing intoxication [226, 227]. The biological and behavioral responses of zebrafish to alcohol are similar to that of humans. Moreover, because they are highly social, zebrafish is an ideal model for studying the physiological and sociological effects of alcoholic foods on humans.

#### 4.2.2. Opioid

Increased abuse of novel drugs has created a critical need for affordable animal models for studying drug-induced toxicity and its impact on metabolism [228]. Although opioids, which are used to treat neurological diseases, have therapeutic effects, they may trigger multiple adverse effects if abused. Psychotropic drugs can cause behavioral changes by affecting nervous tissues and interacting with molecules *in vivo* [229]. Because zebrafish possess an evolutionarily conserved opioid mechanism of action that is also found in mammals, they are suitable models for phenotype-based drug discovery [230].

Morphine is a typical example of addictive opioids. Exposing adult zebrafish to morphine at 2 mg/L for 15 min induces acute morphine addiction [231]. Hydrocodone hydrochloride, a morphine derivative, induces high drug dependence. Zebrafish exhibit continuous drug-seeking behavior after drug withdrawal even if the drugs also cause anxiety and tension [232]. A study on the relationship between *mir-133b*, the dopaminergic system, and morphine using zebrafish identified the higher *mir-133b* levels as a mechanism for the development of addiction to drugs that increase dopaminergic levels in the extracellular space, highlighting *mir-133b* as a potential therapeutic target against addictive disorders [233]. New psychoactive substances (NPS), also called “designer drugs,” are rapidly emerging in illicit drug markets [234]. Studies have identified the early stage of zebrafish development as a promising stage for the early screening of single or mixed NPS, thereby rapidly generating data on their toxicity, behavioral effects, and metabolism [235–239]. A comparison of opioid toxicity and behavioral responses in zebrafish versus rodents has highlighted zebrafish as a suitable alternative model for rapidly testing synthetic opioids.

To measure drug-seeking behavior and gain insight into the underlying biological pathways, Gabriel et al. developed an automated opioid self-administration assay in zebrafish and found that conditioned fish continued to seek the drug despite its adverse consequences and showed signs of stress and anxiety upon drug withdrawal [232]. With its simple and efficient method, this assay has the potential to facilitate the identification of crucial pathways that govern drug-seeking behavior. This, in turn, may pave the way for the discovery of novel molecules that could be used to treat addiction.

#### 4.2.3. Cannabinoids

Cannabinoid use has markedly increased in recent decades, and studies in zebrafish have recapitulated its effects in human addicts [240]. The psychoactive component in cannabis is Δ9-tetrahydrocannabinol (Δ9-THC), while its nonpsychoactive component is cannabidiol (CBD). Exposing zebrafish embryos to Δ9-THC or CBD for 5 h revealed that Δ9-THC negatively affects their motor ability and that both compounds reduced the number of neurons, while reducing heart rate reduction and causing heart malformation. High levels of the psychoactive compound, ayahuasca, cause developmental defects and embryo incubation delay in zebrafish [241].

Zebrafish are ideal animals for establishing models for studying addiction and withdrawal behavior due to their suitability for various behavioral analyses. Through these analyses, researchers have been able to gain a better understanding of the complex mechanisms underlying these behaviors [72, 229].. Thus, zebrafish has shown great potential as an effective and affordable screening model for the identification of compounds that counter the effects of drugs.

#### 4.2.4. Nicotine

Nicotine, the main psychoactive compound in tobacco, is the most widely used drug of abuse. Nicotine activates nicotinic (nAChR) and muscarinic (mACHR) cholinergic receptors and promotes the release of a variety of neurotransmitters, including DA, NE, ACH, Glu, and GABA [242]. Studies have shown that the acetyl cholinergic receptor circuits of zebrafish are similar to those of mammals. Eight nAChR subunits have been identified in the nicotine-activated neural circuitry of zebrafish [243, 244]. This review summarized the zebrafish treated with nicotine to illustrate the effects of nicotine on the tested fish. First, some doses of nicotine boosted the courage of zebrafish [60] in a study that administered nicotine at three doses, acute high dose, acute low dose, or chronic low dose, and then used the novel object test to evaluate the boldness of zebrafish. This analysis found that acute and long-term nicotine exposure enhanced and reduced zebrafish boldness, respectively. Secondly, nicotine causes anxiety in zebrafish [245]. Zebrafish exposed to nicotine at 50 mg/L and 100 mg/L revealed that the nicotinic antagonist, UFR2709, reduced nicotine-induced anxiety [246]. Thirdly, nicotine also impacts memory and learning. A study using the *T*-maze task analysis found that nicotine had procognitive effects at doses of 0.02 mg/kg and 0.002 mg/kg, i.p.) in zebrafish, surprised with a visible inverted U-shaped dose-dependent effect [247]. Indicating that various nicotine doses affect memory. Previous studies found that the administration of low nicotine doses can improve memory [248]. Fourth, nicotine impacts addiction and social behavior. Several studies have shown that zebrafish addiction to nicotine is similar to that of mice and humans [60, 249, 250]. Social behavior analysis showed that nicotine loosened previously concentrated schools of zebrafish. Nicotine bitartrate (4 mg/L and 8 mg/L) affects shoaling behavior by increasing the distance between fish, decreasing their swimming speed, and disrupting their polarization [251].

Using zebrafish models to evaluate the effects of nicotine on CNS function will not only improve the treatment of nicotine addiction but also improve our understanding of the molecular basis of nicotine addiction. This suggests that if we can observe brain changes before and after chemical changes and determine which neurotransmitters are involved and where neural adaptations occur, we can identify drugs with the potential to counter such alterations.

#### 4.2.5. Cocaine and Other Drugs

A study back in 2001 found that zebrafish are particularly fond of cocaine [72] and subsequent studies have shown that as in humans, fish's “fondness” for cocaine is heritable. This study has also found that zebrafish exhibit a compulsive need for drugs, which also occurs in people with addiction. Caroline Brennan's team at the Queen Mary University of London found that zebrafish tolerate being chased with nets if this is associated with getting cocaine [252]. A study by Parker et al. tested various drugs, including opioids to learn about the addictive potential of these drugs and found that zebrafish liked almost all the drugs tested, except THC [253]. A 2016 study found that zebrafish larvae exposed to cocaine (0 mg/L, 2.5 mg/L, 5 mg/L, 10 mg/L, and 20 mg/L) exhibited a dose-dependent heart rate response that persisted for 24 h after drug cessation [254].

Together, these studies indicate that zebrafish can fill the gaps in the study of addiction in mammals. Although the addiction index, CPP, and drug recurrence are important indicators of substance-induced mental disorders in human, there is limited data on the use of these measures in zebrafish as a model organism. Zebrafish models have been successfully used to assess the physiological effects of nicotine and its underlying molecular mechanisms on a wide range of complex behaviors, including aggression, anxiety, long and short-term memory, object discrimination, and color preference. Utilizing zebrafish as a model organism to investigate the effects of nicotine on CNS function can offer valuable insights for improving drug addiction treatment, identifying new drug candidates, and enhancing our knowledge of the molecular mechanisms involved in drug addiction [255].

### 4.3. Depression

**Figure 6:**
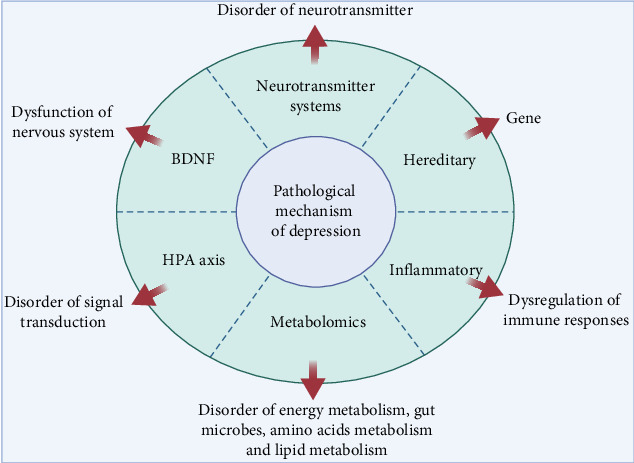
Pathology for different mechanisms of depression. The pathology of depression falls into six categories, including disorder of the HPA axis, abnormal of BDNF signaling pathway, disorder of neurotransmitter, depression caused by genetic or genetic mutations, and metabolomics or disorders of the immune system caused by some diseases (such as metabolic diseases, disorder of energy metabolism, gut microbes, amino acids metabolism, and lipid metabolism), which are complicated by depression. Reproduced with permission from [261].

Depression is a major type of mood disorder that is characterized by significant and persistent depressive symptoms [256]. Severe depression can lead to limb stiffness, which may result in aggressive locomotion and delusion [257]. Like many kinds of mental disorders, depression is recurrent. Although each bout of depression can be relieved, symptoms can be residual, causing chronic disease [258]. Based on genetic characteristics, depression is classified as primary or agitated. Primary depression does not have any clinical manifestations before onset, while agitated depression exhibits psychokinetic excitement, as seen with emotion depression [259, 260]. There are many hypotheses about the pathogenesis of depression (Figure 6), and these mechanisms are related to each other and contribute to the development of depression.

Earlier zebrafish models of depression were constructed using mammals [53]. Behavioral analysis of depression and a variety of depression indicators, such as cortisol, dopamine, serotonin, NE, GABA, and 5-HT [14, 262, 263], are important prerequisites for the establishment of zebrafish models of depression. For example, mammalian CUMS has been modeled in zebrafish [264]. The combination of CUMS and developmental isolation was used to construct a zebrafish model of depression [264]. The sociological behavior of depressed zebrafish was analyzed using NTT [51]. Here, the metabolic levels of dopamine and serotonin in zebrafish brain were significantly reduced. NTT and social behavior tests can also simulate human depressive behavior. These behavioral tests also reflect the indirect memory reduction caused by depression [265, 266].

In recent years, drug-induced depression models have emerged [67]. Reserpine, one of the side effects, leads to mental weakness and delayed behavior and has been widely used to develop mammalian models of depression [267]. An analysis that cocultured embryos (6 h after fertilization) in reserpine [268] and subjected juvenile zebrafish to behavioral tests on day 5 found that movement distance and frequency were shortened, which is consistent with similar observations in a mammalian model of depression, and also decreased levels of dopamine and serotonin metabolism. Exposing adult zebrafish to 40 *μ*g/mL of reserpine for 20 min/day also triggers depression-like behavior [67].

Gene-level development of model organisms also has high success rates [269]. Human genes associated with the HPA and hypothalamic-pituitary-thyroid (HPT) axes are highly conserved in zebrafish. However, the mechanisms by which depression affects these endocrine pathways are not fully understood [270, 271]. Furthermore, HPA defects and neuroinflammation (associated with depression in humans) can be detected by measuring stress-related hormones (such as cortisol) and proinflammatory and anti-inflammatory biomarkers. The zebrafish GR gene, which expresses a receptor protein for the HPA axis [272], establishes a depression model [55], which exhibits HPA hyperactivity [273].

Despite advances in the development of zebrafish models of depression, there are important differences between zebrafish and humans. For instance, there is no way to study placebo effects in zebrafish and other animal models, which are prevalent in the treatment of human depression. A recent study showed that there are gender differences in the contribution of the bilateral caudate nucleus and posterior cingulate gyrus to depression [274]. Differences in abnormal resting-state brain activity between male and female patients with major depression disorder (MDD) were observed in the bilateral caudate nucleus and posterior cingulate gyrus. Moreover, in female MDD patients, the average low-frequency amplitude of the right caudate nucleus positively correlated with the disease course. However, this study did not identify a link between depression and the women's living environment or stress. For this, a large zebrafish sample size should be adopted to study sex differences in abnormal resting-state brain activity.

### 4.4. ASD

**Figure 7:**
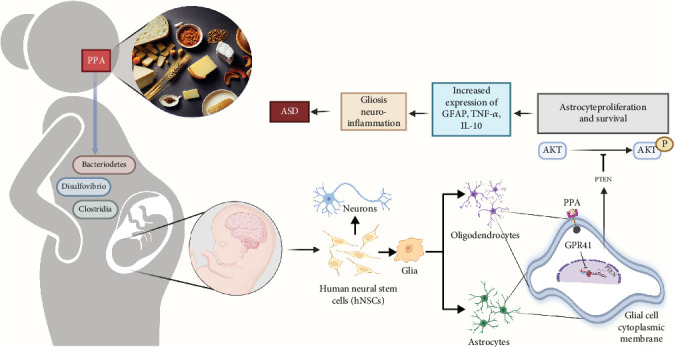
PPA participates in the ASD mechanism route. Excessive PPA combined with preexistent dysbiosis in the diet may interfere with fetal neuronal differentiation in early pregnancy. Here, PPA binds to the GPR41 receptor preferentially expressed on glial progenitor cells and participates in the interference process. Furthermore, changes in downstream pathways including inhibition of PTEN and activation of the prosurvival AKT pathway ultimately lead to the proliferation and differentiation of glial progenitor cells. Here, mature glial cells produce inflammatory cytokines (such as GFAP, TNF-*α*, and IL-10), and large amounts of gliosis promote neuroinflammation, leading to ASD. Reproduced with permission from [276].

Autism, which is caused by defects in neurodevelopment, belongs to one of the major personality disorder types that affect about 2% of people worldwide, most of whom are children or adolescents [275]. Autism is a major medical and social problem that urgently requires attention. A successful organism model is key for deciphering the relationship between the CNS and autism. Various ASD-associated genes have been identified by conducting studies in rodents and humans. However, the processes by which these specific genetic variants lead to behavioral disorders that characterize this disease remain elusive. Although the pathogenesis of autism has not been definitively proven, excessive propionic acid (PPA) combined with preexistent dysbiosis in the diet may interfere with fetal neuronal differentiation in early pregnancy (Figure 7) [276]. Clinical manifestations of autism include resistance to emotional contact with others, accompanied by some bizarre, repetitive behaviors [277], and is mostly hereditary [278]. Environmental stimuli during pregnancy can also lead to autism in the fetus. Males have a higher probability of developing autism than females, which may be attributed to the fact that autism-associated genes are mostly concentrated on the Y chromosome [279]. There are also comorbidities in autism [280]; thus, zebrafish models of autism have to be described based on core indicators of autism. Zebrafish are ideal models for conducting ASD drug screening, identifying environmental risk factors affecting ASD, and exploring potential therapeutic approaches.

Variants and deletions of *cntnap 2* are strongly associated with several mental disorders, including autism [281]. *cntnap 2* interfered with the functions of inhibitory neurons, which triggered autism-related symptoms; however, these symptoms can be alleviated by estrogen analogs in zebrafish [282]. Besides, *shank-1*, *-2*, and *-3* are also associated with autism [283]. *Sam3*^−/−^zebrafish show three core ASD phenotypes, including impaired responses to social novelty, social communication deficits, and repetitive behaviors. Through behavioral tests and assessment of chemokine-related protein levels, the sex-differentiated nature of ASD has been reported [284, 285]. Knockdown or disruption of *syngap1* or *shank 3* expressions in zebrafish affected the early development of the midhindbrain region of the brain and led to hyperexcitable behaviors, which points out the exact timing and location of these genes affecting brain development and function [286]. Furthermore, *dyrk1A*^−/−^ zebrafish showed autistic traits [287]. Neuronal activities of the brain in *dyrk1A*^−/−^ zebrafish were investigated by labeling stress response-related molecules (*c-fos* and *crh*) followed by *in situ* hybridization to validate model building. Expressions of *c-fos* and *crh* in hypothalamic regions were suppressed, relative to those of the control group, indicating that zebrafish were less influenced by the social environment after *dyrk1A* knockout. Knockdown of the autism-related gene (*shank3b*) in zebrafish [288] was associated with a significant reduction of presynaptic- and postsynaptic- related proteins (*homer1* and *synaptophysin*). Thus, *shank3b* is involved in ASD. The above validation methods at the gene level included behavioral analyses to further characterize their reliability [284, 285, 287, 288]. The oxytocin system is directly correlated with autism development. With regards to the oxytocin receptor-related gene (*oxtr*), the zebrafish lacking the oxytocin receptor exhibited changes in aggressive, social- and anxiety-related behaviors and showed sex differences in males over redundant females [289]. Other genes, including *nrxn*, *ngln*, and *synap1b* in zebrafish are also associated with autism. Model organisms have also been studied and successfully used for ASD drug screening [290, 291].

Zebrafish ASD models can also be constructed by drug induction. Valproic acid (VPA) affects *shank3* expressions and can alter the establishment of *shank3* isoform-induced ASD models [292]. Moreover, VPA induced damage to neural development in zebrafish. Embryonic exposure to 75 *μ*M VPA at 4-5 h postfertilization, which was validated at the molecular level and behaviorally analyzed from day 7 until day 21, was associated with ASD-like phenotypes in zebrafish, exhibited biochemical and cellular levels that were comparable to those of rodent models in terms of drug screening, such as experimental designs of drug targets [293].

Autistic zebrafish exhibit low social acceptability, while 90% of healthy zebrafish exhibit shoaling behaviors [44]. In addition to neurotoxins such as VPA, heavy metal ions such as Pb, which are present in large quantities in crude oil pollutants, can also induce ASD symptoms in zebrafish. According to a previous study, exposure to Pb or crude oil contaminants can alter the original shoaling behavior of zebrafish, resulting in dispersed ASD-like behavioral phenotypes [45].

### 4.5. Schizophrenia

**Figure 8:**
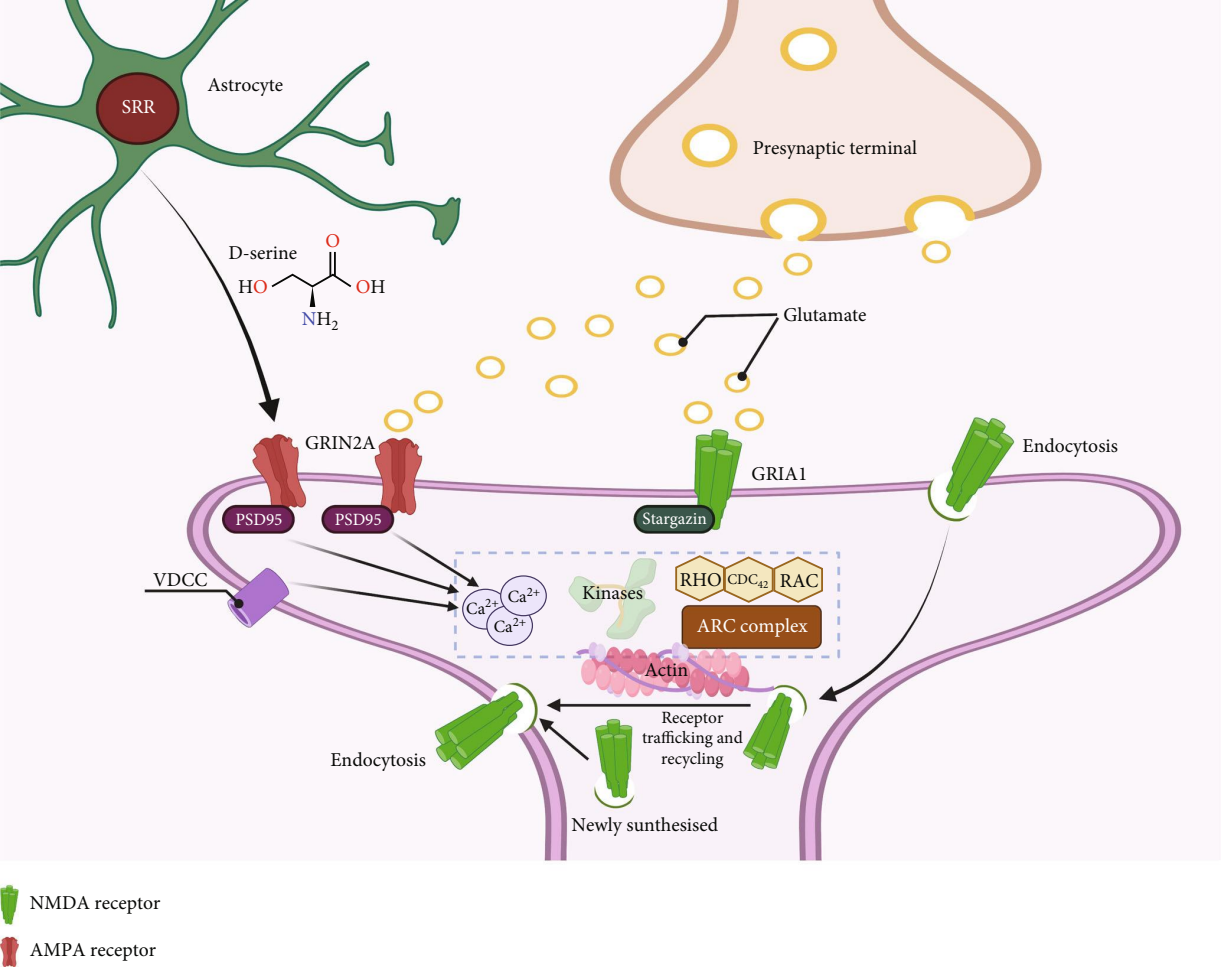
Mechanisms of schizophrenia-regulation of glutamate synapse. Glutamatergic neurotransmission plays an important role in schizophrenia. Schizophrenic-related genes in the glutamatergic neurotransmission pathway include GRIN2A, GRIA1, SRR, CACNA1C, genes encoding the ARC complex, and other several genes encoding schizophrenic-related proteins. The NMDA-type glutamate receptor is modulated by D-serine, a coagonist synthesized by SRR. VCDDs are some proteins encoded by CACNA1C, and they are involved in the regulation of intersynaptic signaling through intracellular calcium channels. In response to glutamate receptor activation, proteins associated with postsynaptic scaffold aging (including PSD95, CACNG2, several kinases, RHO, CDC42, RAC family of small G proteins, and ARC complex) are mainly involved in intracellular signal transduction. Here, the square dotted lines are related proteins that determine the basis of synaptic plasticity. Reproduced with permission from [295].

Schizophrenia, a chronic disease of unknown cause, appears in late adolescence to early adulthood. The condition is recurrent and is often accompanied by cognitive decline and emotional disorders [294]. Currently, the more representative pathogenesis (Figure 8) of schizophrenia is the fine-tuning of the glutamate synapse [295]. Schizophrenia has various signs, including positive symptoms (e.g., delusions and hallucinations) and negative symptoms (e.g., aggressive behavior) [59], and is mostly caused by a combination of genetic and environmental factors [296]. Zebrafish reach sexual maturity at three months of age, and hence, they can be used to develop schizophrenia models at 3-10 dpf [297, 298], which may be associated with incomplete BBB development. N-Methyl-D-aspartate (NMDA) receptor inhibitors, including dizocilpine (MK-801), ketamine, or phencyclidine are mainly used to generate zebrafish models of schizophrenia [299].

An analysis of MK-801-induced schizophrenia in zebrafish suggested that schizophrenia is physiologically associated with dopaminergic hyperactivity and loss of GABAergic interneurons, NMDA receptor hypofunction, and redox disorders [300]. MK-801 disrupts social interaction in zebrafish, which is similar to negative symptoms of schizophrenia. At the same time, zebrafish showed significantly more hyperactivity in the presence of other fish than when tested alone. Moreover, at 2 mg/L, ketamine can increase aggressiveness in zebrafish [301], mimicking negative symptoms of schizophrenia. Interestingly, mirror-biting tests revealed that high amiodarone concentrations reduced aggressive behavior in zebrafish.

Moreover, numerous genes are associated with schizophrenia, including *DISC1* (schizotypal schizophrenia), a typical schizophrenia risk gene and also one of the most studied schizophrenia-associated genes, besides *NRG1*, *TH*, and *shank3* [288, 302, 303]. Studies have shown that *DISC1* helps to maintain the structural stability of hypothalamic progenitor cells, normal neuronal differentiation, and the function of the hypothalamic-pituitary-adrenal (HPI) axis in young and adult zebrafish [302].

Because of associated comorbidities, only a few studies have developed zebrafish models of schizophrenia. However, comorbidity is also a feature of human schizophrenia. The lifetime prevalence of substance abuse in schizophrenic patients is 30%–50%, three times higher than in the general population. Patients' substance abuse behavior may precede the emergence of mental symptoms. Moreover, it may aggravate mental symptoms, interfere with a schizophrenia diagnosis, and affect the success of treatment. The rate of smoking among schizophrenic patients is 30%–60%, about 2–4 times that of the general population [304]. MRI analysis revealed significant changes in the binding of glutamate receptor subtypes to some brain regions of schizophrenia patients, such as decreased glutamate receptor expression in the hippocampus and increased expression of glutamate receptor subunits in the cortex [305]. Schizophrenia is associated with various abnormalities in glutamate neurotransmitters. For example, a glutamate marker is decreased in the medial temporal lobe area and postsynaptic glutamate receptor density is increased in the frontal area, suggesting that the central function of glutamic acid may underlie schizophrenia. Finally, various drugs and drug targets have been shown to treat schizophrenia in zebrafish [306–308].

### 4.6. HD

**Figure 9:**
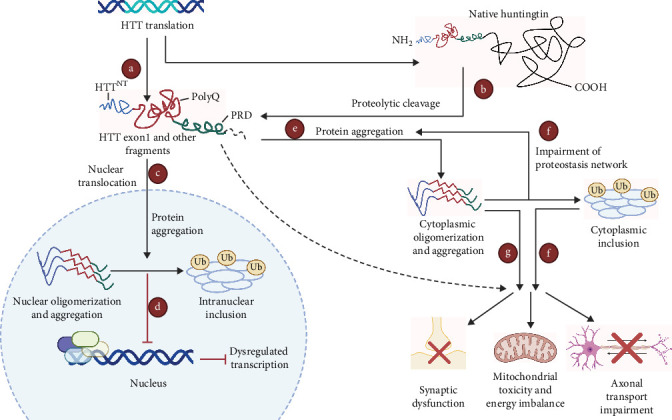
Pathogenetic mechanisms of Huntington's disease. (a) Huntington's disease is an inherited neurodegenerative disorder, associated with an expanded polyglutamine (poly Q) region in the protein encoded by the huntingtin (HTT) gene. (b) In response to proteolytic enzymes, the full-length native huntingtin protein is cleaved to produce additional protein fragments. (c) HTT exon1 and other fragments are transferred to the nucleus. (d) After aggregation, these proteins format inclusions in the nucleus. (e) Huntingtin fragments oligomerize and aggregate. (f) Disease-related disruption of the proteostasis network accelerates huntingtin aggregation. (g) The aberrant of huntingtin results in cellular impairments, including synaptic dysfunction, mitochondrial toxicity, and a decreased rate of axonal transport. Reproduced with permission from [310].

HD, also referred to as Huntington's chorea, is an inherited neurodegenerative disorder that is associated with an expanded polyglutamine (poly Q) region in the protein encoded by the *huntingtin (HTT)* gene (Figure 9) [309, 310]. Mutations of the *HTT* result in the accumulation of mutant proteins in the brain, which are mainly aggregated in the striatum. As the disease progresses, the patient gradually loses the ability to speak and move, accompanied by cognitive decline [311]. There are decreased activities of the mitochondrial oxidative respiratory chain and iron-stable defects in the striatum of HD patients [312–314]. These defects are expected to improve the establishment of animal models in the future.

The *HTT* gene has a major role in HD pathology [315]. In zebrafish, complete abrogation of expressions of *HTT* resulted in embryonic lethality [316]. Schiffer et al. [317] reported that HD is caused by repeated expansion of the CAG (encoding glutamine) trinucleotide. *HTT* converts into an unusually long polyglutamine bundle. The overexpression of the polyQ protein may contribute to severe phenotypes of HD disease. *mHTT-exon1* is an HD model of zebrafish, while zebrafish with intact *N17* and *97Q* expansion [318]. Deletion of *HTT* exon N-terminal 17 amino acids (*N17*) enhances motility, implying that *N17* affects the stability of polyQ. A recent study showed that cGMP, through protein kinase G (PKG), activates 26S proteasomes and enhances protein degradation. Inhibitors of phosphodiesterase 5 affected polyQ aggregation in HD zebrafish models by regulating cGMP levels *in vivo*. Moreover, increasing cGMP or cAMP levels reduced HD pathology in zebrafish models [111].

Zebrafish showed behavioral changes that were attributed to long-term 3-nitropropionic acid (3-NPA) treatment. Zebrafish showed increased heart rates and reduced exercise capacities after 3-NPA exposures. Motor deficiencies are not limited to adult and larvae forms [319]. Quinolinic acid- (QA-) induced CNS disorders have been reported in zebrafish, manifesting as HD and stork [320]. QA stimulates robust regeneration of adult neural stem cells, with long-distance integration of new neurons in adult zebrafish. This study provides new insights into the mechanisms involved in mammalian nerve damage.

In general, HD zebrafish models have commonalities with mammals, such as overexpressions of mutant *HTT*. However, there is a need to elucidate on motor phenotypes and responsiveness to drug treatment using these models. The symptoms of HD, such as irritability and depression suggest that we can use similar behavioral approaches to establish zebrafish HD models. Distinctively, combined with biomarker-level analysis, a more standard model can be obtained.

### 4.7. Other Types of Mental Disorders

#### 4.7.1. ADHD

**Figure 10:**
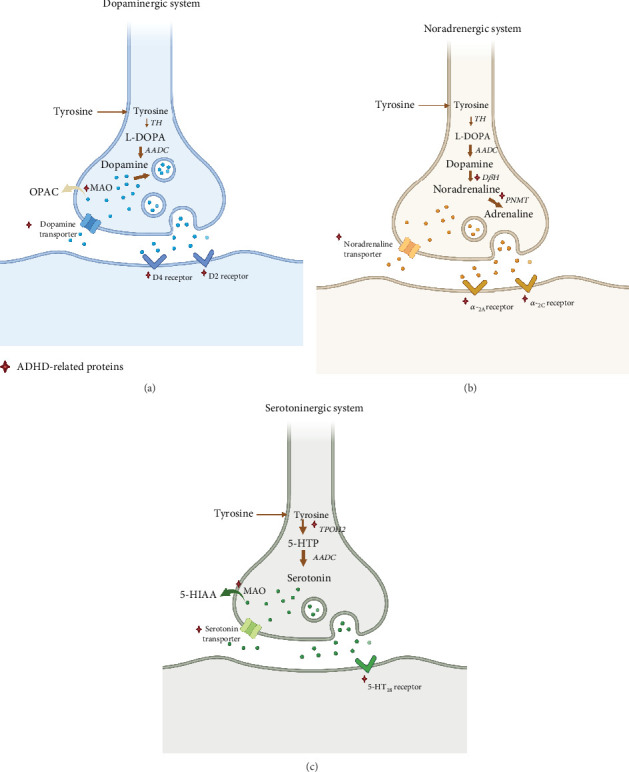
Major ADHD-related proteins and their association with the brain monoaminergic systems. (a) Dopaminergic system. (b) Noradrenergic system. (c) Serotoninergic system. Reproduced with permission from [322].

Patients with ADHD present with inattentive or short attention spans, depressed moods, or impulsive behaviors. It is a syndrome in which multiple states coexist and most patients are children [321]. Currently, it is generally postulated that the pathogenesis of ADHD is driven by genetic factors, which are the basis of the pathogenesis. Moreover, ADHD is related to the abnormal metabolism of neurotransmitters in the brain, which may be related to the dysfunction of the metabolism of the neurotransmitter dopamine system (especially NE) (Figure 10) [322]. *sapap4*, which has an important role in synapses, is associated with ADHD [323]. A recent study revealed that *sapap4* may play an important role in synapses and be associated with ADHD [324]. ADHD has also been reported in adults, and patients are primarily associated with emotional frustrations, which manifests as inattention states [325, 326]. An international study involving more than 50,000 people worldwide revealed genetic overlaps between ADHD and diseases such as depression, anorexia, obesity, reproductive functions, smoking, or insomnia, and 75% of disorders can be explained by genetic factors [327]. This study further suggested that ADHD is a disease with a solid biological basis and that genetics is significant in this regard. Altered circadian rhythms can affect DA levels in zebrafish, resulting in ADHD-related phenotypes. Therefore, inhibition of circadian-related homologs (*micall2b* and *lphn3.1*) triggers impulsive behaviors in zebrafish with saturated dopaminergic neurons [328, 329]. In addition, *period1b^−/−^* zebrafish exhibit ADHD-like symptoms, including hyperactivity, impulsivity, attention deficits, and lower dopamine levels [7, 330, 331]. Furthermore, *lphn3* is involved in synaptic neurotransmitter release, as well as neurodegeneration during ischemia and hypoxia. Recent research suggests that lphn3 may be involved in a novel neural pathway that acts as a susceptibility factor for ADHD both in childhood and adulthood. This highlights the potential importance of this genetic marker in the development and persistence of ADHD [332]. *lphn3.1* is the homolog of *lpn3* during zebrafish development. Inhibition of *lphn3.1* in zebrafish using morpholino resulted in hyperactive and impulsive phenotypes and reduced and misplaced dopamine neurons in the ventral interbrain [333].

Comorbidities limit the establishment of ADHD animal models. Inattentiveness and depression are also characteristics of other mental disorders. Moreover, due to individual differences, ADHD is associated with varying degrees of impulsive or inattentive behaviors. There are no specialized behavioral analysis methods for accurate quantitative characterization ADHD behavior. However, given that HD is mainly genetically inherited, zebrafish has great advantages over other mammals in the development of gene editing technologies; characteristics such as *in vitro* fertilization and high sample sizes, transparent embryos, and genome are easily modifiable.

#### 4.7.2. ALS

**Figure 11:**
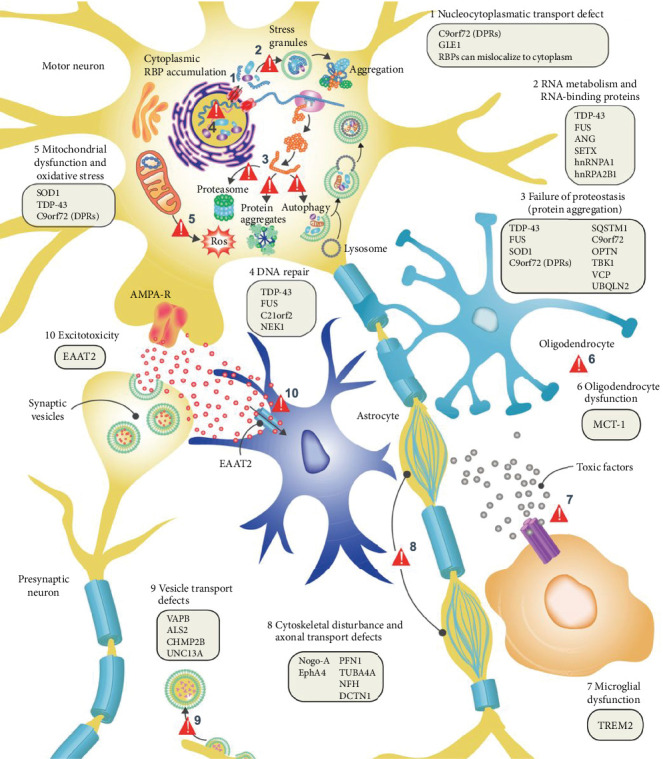
ALS disease pathology at the cell level and different mechanisms of motor neuron degeneration. (A) RNA transport from the nucleus to the cytoplasm. (B) Motor neuron degeneration, including axonal contraction and cell body loss of upper and lower motor neurons, and surrounding astrogliosis and microgliosis are the main pathological features of ALS. Changes in RNA metabolism, such as dysfunctions of transcription and splicing defects. (C) The continuous accumulation of TDP-43, FUS, SOD1, and DPRs results in impaired protein balance. (D) Impaired DNA repair. (E) Increased mitochondrial dysfunction and oxidative stress. (F) Dysfunction and degeneration of oligodendrocyte, damage neurons. (G) Generation of neuroinflammation. (H) Abnormal axonal transport. (I) Defective vesicular transport. (J) Excitotoxicity. Reproduced with permission from [350].

ALS is a progressive neurodegenerative disease that affects motor neurons in the brain and spinal cord [334]. As the base of people with mental illness increases, the number of people with ALS is increasing year on year, and there are clear regional differences, a trend toward higher prevalence in Europe than in Asia [335]. There are no effective treatments for this condition, and patients mainly present with severe skeletal muscle weakness that characterizes a motor neuron disease [336]. The etiology of ALS has not been fully established; however, ALS can be neurotoxic and genetically determined (Figure 11) [337]. The zebrafish is a particularly attractive model to study the function and dysfunction of spinal cord circuits because of its transparent vision during the early stages of life and the high anatomical and biological functional similarities between the zebrafish and human spinal cords.

There is a direct link between the heavy metal ion of Pb and ALS, mainly in the form of reduced acetylcholinesterase activities without altering gene expression patterns. In zebrafish exposed to 20 *μ*g/L mercuric chloride and lead acetate, brain acetylcholinesterase activities were assessed at 24 h, 96 h, and at 30 days postexposures [338]. Other neurotoxic drugs, including *β*-N-methylamino-L-alanine (BMAA) and bisphenol A (BPA) can induce ALS-like phenotypes in zebrafish [339]. Zebrafish exhibit ALS-like behaviors when exposed to BMAA, with memory deficits accompanied by disruptions in neurodevelopment [340].

The ALS-associated genes include *TARDBP*, *FUS* [341–343], and *C9orf72* [344], among which *C9orf72* mutations ar common in ALS patients. Hexanucleotide repeat expansions within the *C9orf72* gene are the most common genetic cause of ALS [345]. A previous study [346] showed the toxicity of poly-GA to zebrafish. The GGGGCC repeat expansion in *C9orf72* has been proven to be associated with ALS pathogenesis [344]. Another model construction method involves specific expressions of GR dipeptide repeats (*DPRs*). When GR *DPRs* are specifically expressed in motor neurons, developmental defects are significantly reduced, but swimming phenotypes persist, suggesting that GR *DPRs* have toxic effects on motor neuron functions [347]. In addition, *TDP43-A315T* and *G93R-Msod1* zebrafish are potential ALS models [348]. Moreover, mutations in fused in sarcoma (*FUS*), an RNA-binding protein, and depletion of *FUS* homolog in zebrafish models are associated with the main physiopathological features of ALS, including impaired motor abilities and shortened motor neuron lengths [349].

In conclusion, *C9orf72* plays an important role in ALS. In zebrafish models, attention should be paid to risk detection of comorbid disorders caused by ALS. At the same time, the construct validity of gene expressions should be elucidated further. In recent years, other ALS-associated genes have rarely been reported. These genes include *FUS*, *SOD1* (the first gene believed to be associated with ALS, containing multiple mutations), and TAR-binding protein 43 (*TDP-43*). Behavioral breakthroughs of ALS in zebrafish are mainly associated with a reduction of swimming endurance; however, the use of zebrafish in ALS studies is associated with some limitations, including a lack of upper motor neurons, widespread use in embryos, and a lack of zebrafish antibodies for research [351].

### 4.8. Application of Zebrafish Models in Psychiatric Complication

Human with severe mental disorders, including schizophrenia, bipolar disorder, and other nonorganic mental disorders, have an increased prevalence of a range of chronic conditions. Understanding the physical conditions that are commonly present before and after a diagnosis of serious mental disorders is crucial. This can allow researchers to obtain necessary timing information for administering preventive or therapeutic interventions.

Zebrafish models provide an important platform to study mental disorders. In previous studies, zebrafish was used to investigate diabetes complicated with mental illness [66, 352, 353]. Here, Zebrafish show higher levels of neuroinflammation and other inflammation related to diabetes. Several other diseases such as cardiovascular disease [354] and chronic inflammation [355] have been to linked to the development of mental disorders. In addition, research indicates that some potential therapeutic targets are applicable for studying the connection between metabolic diseases and aging, such as negative regulator of AMPK activity and GID-complex (evolutionarily conserved ubiquitin-ligase-complex) [356]. It seems that we can effectively accelerate the pace of drug development. For more than 20 years, people with high blood pressure, diabetes, high cholesterol, or obesity were more likely to develop AD. All of these diseases affect the brain and damage blood vessels, leading to stroke. But despite great efforts by researchers, the link between vascular disease in the brain and AD remains unexplained. Surprisedly, a new study has shown that the *FMNL2* gene participates in the development of cerebrovascular disease and AD, suggesting that changes in *FMNL2* activity caused by cerebrovascular disease prevent effective clearance of toxic proteins from the brain, ultimately leading to AD [357]. The findings may offer a way to prevent AD in people with high blood pressure, diabetes, obesity, or heart disease.

According to the study [358], there are 24 chronic diseases associated with mental disorders; this is the first to describe the temporal relationship between diagnosed schizophrenia, bipolar disorder, and other nonorganic psychosis and 24 chronic health conditions in the 5 years before and after diagnosis. The increased odds of multiple disorders at the time of severe mental disorders diagnosis suggest that early intervention in physical health parameters is necessary to reduce morbidity and premature death. Moreover, it also prompts researchers to study animal models of psychiatric complications from these 24 diseases for drug development.

## 5. Challenges and Future Perspectives in Zebrafish Models of Mental Disorders

Although zebrafish has been applied to the construction of a variety of mental disorders models, its limitations cannot be ignored. As is inevitable with other model animals, zebrafish and humans have differences in brain structure and function that may limit the interpretability of the findings under human conditions [359]. Consequently, zebrafish cannot completely replace the clinical research of the disease. Additionally, the behavioral assays used in zebrafish research may not fully capture the complexity of human mental disorders, which needs to be fully verified by multiple types of behavioral analysis methods and caution must be exercised when drawing conclusions based on these results.

Cognition, learning, and memory in zebrafish can bridge the gap between *in vitro* and *in vivo* studies because of its advantages over rodents [262, 360, 361]. Indeed, unlike the phenotypic complexities of mammals, which make it difficult to predict cognitive processes, zebrafish phenotypes facilitate the identification of behavioral forms. Stress is an important factor influencing memory performance in all species. Similar to humans, zebrafish release cortisol as the primary stress hormone, rather than corticosterone as in rodents. Differences between zebrafish, rodents, and humans must be taken into account when designing experiments and interpreting results. Despite several key advantages of zebrafish in learning, memory and cognition studies, limitations should be noted, which are mainly related to morphological and neurophysiological differences between zebrafish and mammals. Notably, zebrafish do not have typical astrocytes, and even if they do contain a large number of radial glia, it is unclear whether astrocyte-like cells play a role in BBB function. Moreover, zebrafish habitats make it impossible to accurately determine drug uptake and utilization by zebrafish or to quantitatively assess them [362].

In light of the issue of controlling dosage in zebrafish, one of our previous studies [352] has proposed a novel approach for determining the appropriate dose. This involves utilizing a medicine and food homologous delivery system in combination with the lyophilization method, which helps to minimize the dispersion of drugs in fish water. Phenotypic analyses of zebrafish, especially complex phenotypes, such as natural variations of animals are challenging, even if these can be done by creating many different research groups. Another challenge involves the production of enough zebrafish to achieve a truly large-scale high throughput screen. Improved methods for mass production and handling of zebrafish embryos would be beneficial. With the latest advancements in gene editing technologies, we have successfully regenerated dozens or even hundreds of human genetic mutations in zebrafish. These genetically modified zebrafish can be utilized for evaluating phenotypes and for screening chemical inhibitors that are associated with various diseases.

Moreover, it was around 2010 that zebrafish began to be used in the academic field. Compared to mammals, there is a long way to go before zebrafish are fully integrated in mental disorders. The use of a single model cannot identify and redirect the pathologic course of mental disorders. Human mental disorders are highly complex and repetitive. Only a comprehensive and comparative review of different model organisms can provide answers to the remaining questions about mental disorders. Although different model organisms have obvious characteristics, only a simultaneous and comparative analysis of these different systems can unravel the causes and mechanisms of mental disorders.

Some behavioral tests in mammals and zebrafish are actually similar in principle, including the Morris water maze sand radial arm maze tests [363]. The sucrose preference test [364] is a measure of anhedonia, which refers to a lack of interest in rewarding stimuli, a form of mood disorders, used to characterize stress-coping strategies in rodent models. The forced swim test [365], a widely used approach in basic research and screening of potential antidepressants, is one of the most commonly used tests to evaluate depression-like behaviors in rodent models. Hence, novel zebrafish behavioral testing methods aimed at obtaining highly consistent indicators of the clinical phenotypes of mental disorders will become a breakthrough to narrow the gap between zebrafish and mammals in the future.

Advantages, such as the extensive use of genome editing techniques in zebrafish, attributed to 87% homology of the genome similar to humans. Zebrafish embryos are transparent and have a short incubation period. This makes genome editing for these embryos easier compared with embryos from other animals. A previous study showed that using CRISPR-Cas9 to edit zebrafish genes can be 6 times more effective compared with other techniques [366]. Gene editing and real-time high-resolution imaging technologies help scientists investigate disease progression at the molecular level and develop accurate disease models. Gene editing in zebrafish can generate models containing human alleles which improve the research into the pathomechanisms driving human diseases, including CNS diseases [367]. Moreover, a large number of transparent zebrafish embryos can be easily edited, genetically modified, and used to screen for drug efficacy. During the embryonic stage, it is easier to explore the biology and function of organs and tissues at a single-cell resolution which allows researchers to investigate the complex processes, including developmental biology, developmental disorder models, stem cell regeneration, and neural circuits [368–370]. Moreover, single cells and tissues can be screened in living embryos using fluorescence reporting systems for visible phenotypic screening.

Nowadays, using zebrafish models to study mental disorders and develop drugs is already a reality [371]. For example, zebrafish with loss of function mutations in *SCN1Lab* exhibit spontaneous electrographic seizures and spastic-like swimming behavior with clinical features of Dravet syndrome in children [372]. A screen of more than 3000 commercially available and FDA-approved drugs identified the 5-HT receptor agonist Clemizole as a chemical inhibitor of spontaneous seizures in *SCN1Lab* mutants that inhibited spasmoid behavior. EpyGenix Therapeutics is currently developing Clemizole (EPX-100) for clinical use and confirmed that EPX-100 was safe and well tolerated in children, and EPX-100 is now proven to be a therapeutic agent. Moreover, the organization and function of the zebrafish dopaminergic system are more stable than that of mammalian vertebrates, making zebrafish a practical and economical animal model for testing the effects of neuroactive compounds ^373^. Here, stavudine, tapentadol, and nabumetone were confirmed as targeted agents for PD drug screening in zebrafish larvae with 6-OHDA injury.

To improve future drug screening using zebrafish models, there is a need to incorporate multiple screening parameters into the whole animal screen. This will provide a more comprehensive understanding of the response to chemical targets and drug therapy, including both distal target and nontarget drug effects. Additionally, the use of single-cell omics techniques will allow for the assessment of the impact of drugs in large cell populations, providing unprecedented detail on the cellular manifestations of disease and how it is resolved. Currently, zebrafish disease models and small molecule screening have yielded exciting results, which bodes well for their potential to make significant contributions to the treatment of human diseases in the future.

## 6. Conclusions

Mental disorders afflict people all over the world and cause enormous personal and social burdens. For a long time, a variety of animal models have made great contributions to a better understanding of the mechanisms of human mental disorders in order to obtain critical information in identifying biological and molecular targets for the development of safer and more effective therapeutic strategies. The zebrafish has emerged as a promising animal model for studying neurobehavioral and neuropsychological phenomena, thanks to its similarity with mammals in terms of key brain regions. This homology underscores the potential of zebrafish models in these research fields. In addition, the conservation of neural pathways between zebrafish and mammals allows bidirectional translation of findings. Current genetic tools, tracking techniques, and statistical algorithms have improved our understanding of the molecular pathways and are expected to enhance the development of new drugs or new applications for old drugs. Combined with the high sensitivity of zebrafish to drugs known to treat mental disorders, zebrafish provides an important platform for investigating the pathogenesis of mental disorders and the development of therapeutic strategies.

## Figures and Tables

**Table 1:** Different behavioral analysis tests of zebrafish in mental disorders.

Category	Behavior tests	Schematic diagram of the devices	Main operating principles	Ref.
Behavioral analysis of basic motor and sensory functions	Swimming test	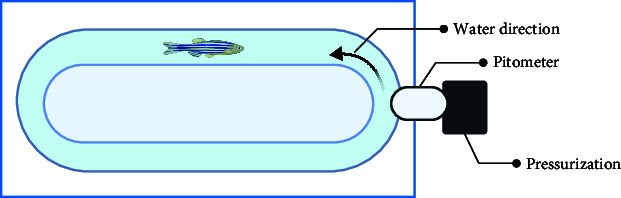	The device contains a pitometer and pressurization. Zebrafish are individually placed into the lane, producing spontaneous movement due to the zebrafish's congenital upstream swimming characteristics. Tested zebrafish will produce spontaneous swimming when the water speed reaches 12 cm/s.	[46]
	Optokinetic response (OKR)	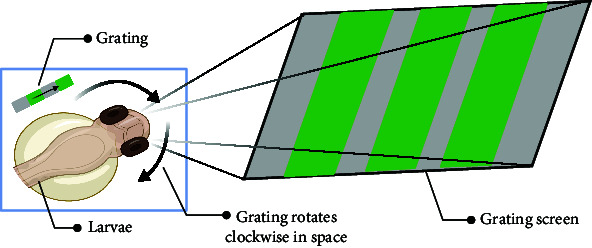	The arena covered by grating stimuli covered most of the zebrafish larvae's visual field. OKR at different stimulus positions, related behavioral performance to photoreceptor densities in the retina.	[47]
	Stress response	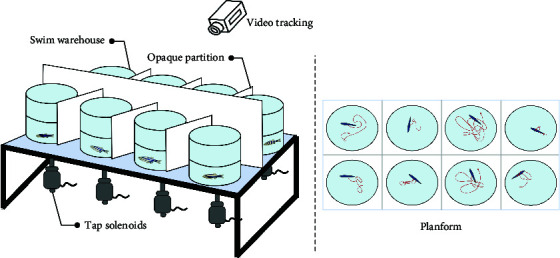	Video is captured from above 8 swim warehouses. Solenoids piston under the arena.	[48, 49]
	Visual conditioned/ unconditioned stimulus	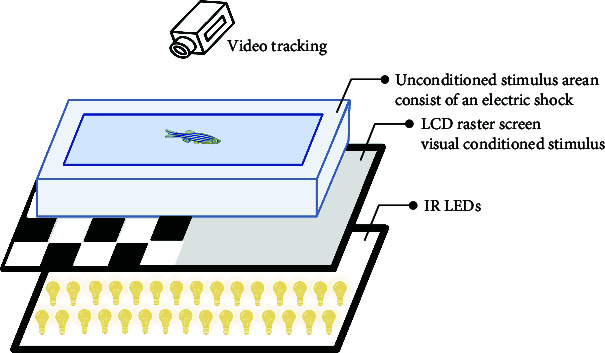	Video is captured from above the arena. A single zebrafish is placed in the arena, LCD screen for visually conditioned stimulus beneath the fish tank. Electric shock for unconditioned stimulus. IR LEDs are used to help capture zebrafish traces.	[50]

Depression-/anxiety-like behavior	Novel tank test (NTT)	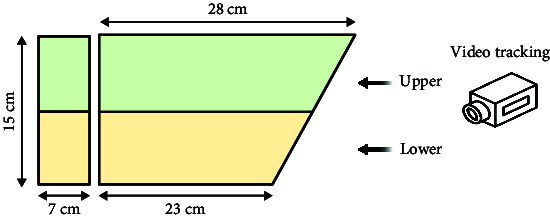	The novel tank consists of two arenas. One baseline in the center of the tank divides it into upper and lower arenas. Video picks up fish tracks from the largest side of the tank.	[51]
	Shoaling test	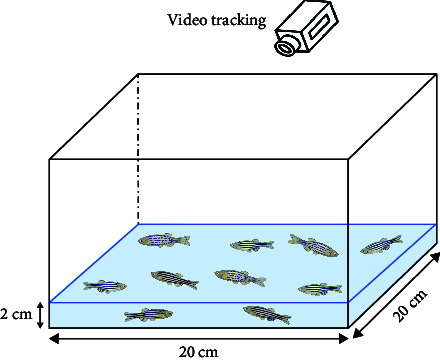	The video is captured from above the arena. Ten zebrafish were involved in each experiment. Depression-/anxiety-like zebrafish will be reluctant to create closer fish distances.	[52]
	Light/dark tank test	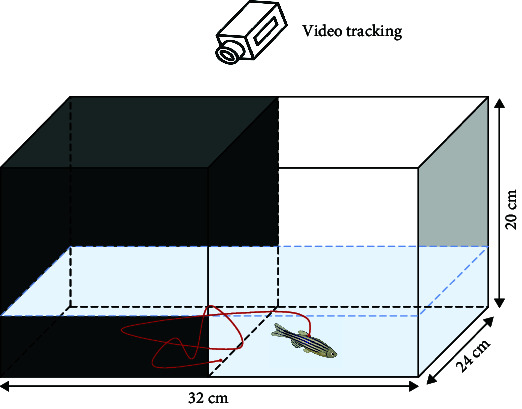	The video is captured from above the arena. The tank is evenly divided into two arenas. One part of the tank is black and opaque, with a partition in the middle and a 2 cm height leaking from the bottom for the zebrafish to pass through.	[53]
	Acoustic startle response	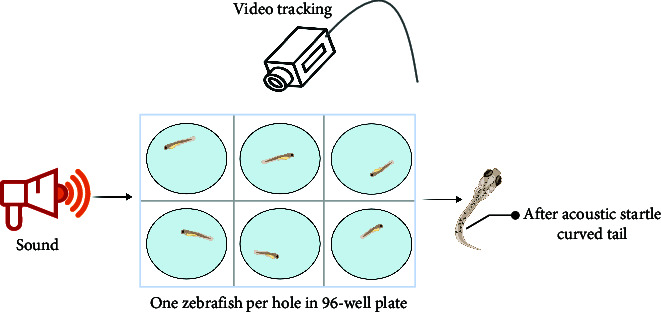	The video is captured from above the arena, video connects to computer for data analysis directly. Zebrafish were put in per hole of a 96-well plate. After acoustic stimulation, the tail of the juvenile fish appears to bend, showing a curved tall.	[54]
	Video-tracking Experiment	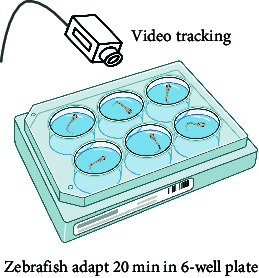	The video is captured behaviors from above the arena, video connects to computer for data analysis directly. Zebrafish were put in per hole of a 6-well plate. These behaviors include edge and downward movement, speed, movement distance, resting frequency, resting average time, swimming speed, turning angle, and distance between zebrafish.	[45]
	Novel object approach test	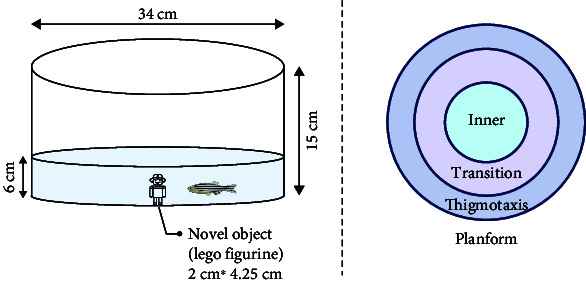	The arena is divided into three sections (inner, transition, and thigmotaxis); there is a Lego man (novel object) put in the inner. Zebrafish were individually placed into the experimental arena.	[55]

Behavioral analysis of learning and memory	Spatial learning test	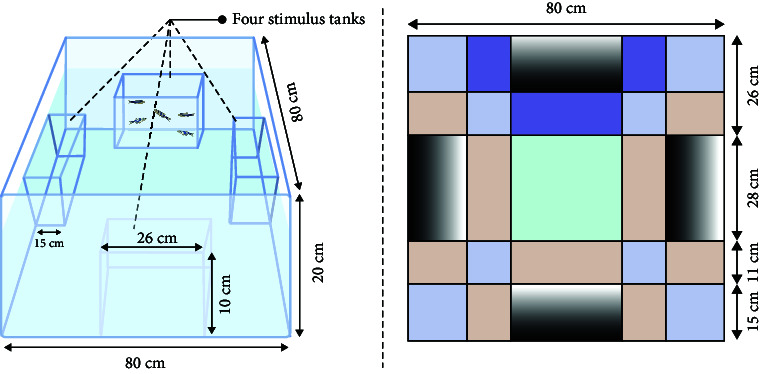	One of the stimulus tanks (black areas) contained five conspecific stimulus fish and was not accessible to the experimental fish. The purple areas represent the target zone that is proximal to the location of the stimulus. The yellow areas represent the proximity zones which correspond to the target zone but are adjacent to stimulus tanks without the stimulus.	[56, 57]
	*T*-plus test	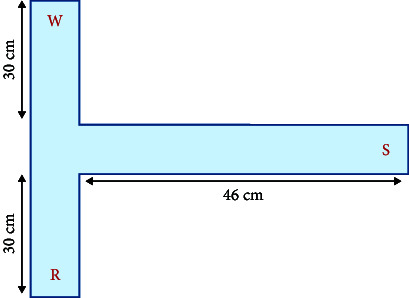	The T-maze is divided into three parts with a 5 cm water depth: (1) “s” area: start arm; (2) “r” area: right area; (3) “w” area: wrong area.	[58]
	*Y*-plus maze	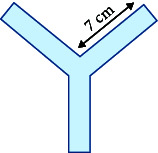	The *Y*-maze is divided into three arms, each arm with 7 cm lane and 1 L aquarium water.	[59]
	Plus maze	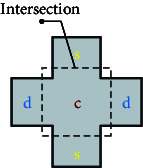	3D and 2D drawings of the plus maze. Maze is divided into five parts, d: deep arm; s: shallow arm; c: center zone, which includes intersection and ramps.	[60]
	Avoidance conditioning	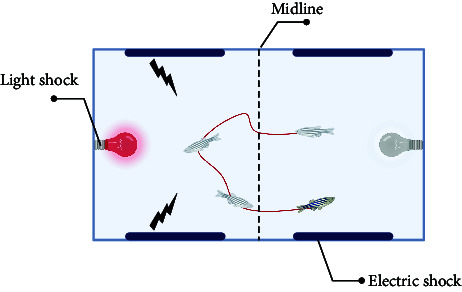	Avoidance conditions including light shock and electric shock. Zebrafish would change direction after a conditioned stimulus.	[61, 62]

Social behavior analysis	Social interaction test	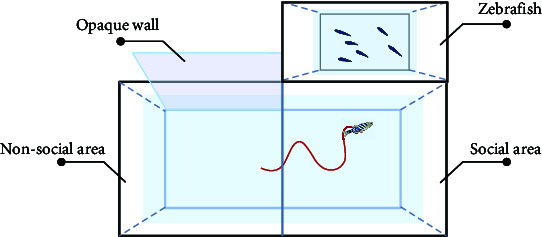	The tank is divided into three regions: nonsocial, social area, and zebrafish room. When the test starts, remove the opaque wall. Determine the frequency and duration of zebrafish activity in two areas.	[58]
	Mirror biting test	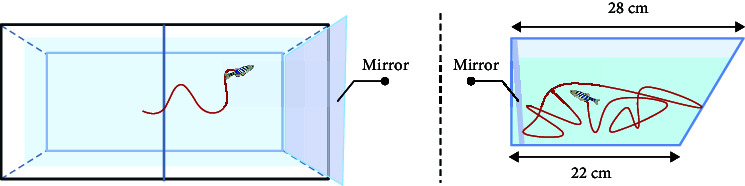	The tank is the same size as NTT but has a tilted mirror on one side of the vertical plane.	[43, 54, 63]
	Shoaling test	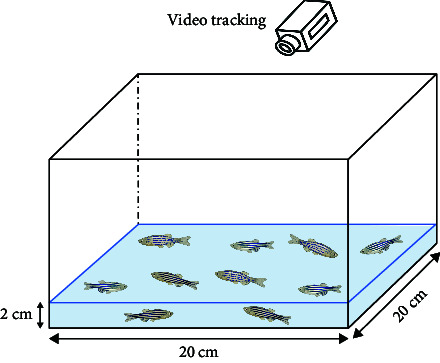	The video is captured from above the arena. Ten zebrafish were involved in each experiment. This test can determine the distance between zebrafish and swimming speed.	[44]
	Predator avoidance test	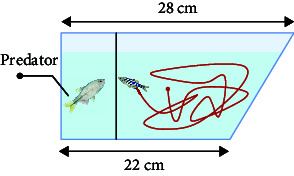	The tank is the same size as NTT but has a predator in one space of the vertical plane.	[63]
	Social preference test	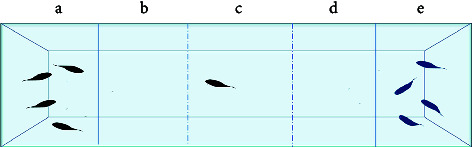	The tank is divided into five regions. (a) e: social stimulus chamber; b, c, d: tested fish chamber; b, d: area of social preference; c: area of no social preference	[64]
	Cluster analysis	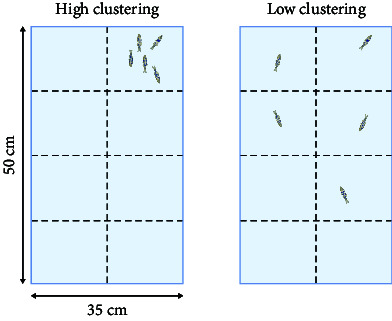	The swarming score at each time point was calculated every 30 s to determine zebrafish dispersion. The formula is to divide the maximum number of zebrafish in a position of the tank by the number of segments occupied.	[65]
	Conditioned place preference (CPP)	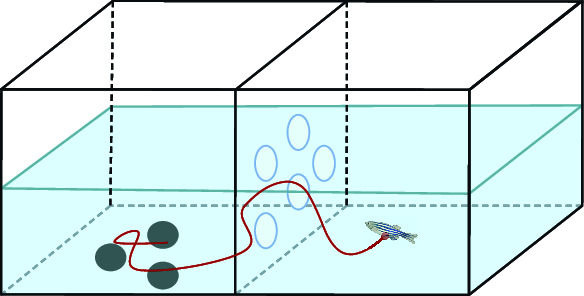	Device can capture preference changes that reflect the effect of giving drug rewards to zebrafish. The tank is divided into two parts, containing three objects of visual cues, and the tank has a perforated wall in the center that allows the zebrafish to traverse freely.	[66, 67]

**Table 2:** Summary of zebrafish mode in CNS diseases.

CNS diseases	Methods	Details	Biomarkers	Ref.
PTSD	Gene editing	*PAC1*	-	[102–104]
		*TPH1*		
		*TPH2*		

AD	Gene editing	*APPsw*	DA	[80, 114, 116, 117, 120, 121, 123–141]
		*A152T-Tau*		
		*PEN1*	NA	
		*PSEN2*		
		*AppA*		
		*AppB*	Dihydroxy-phenylacetic acid	
		*Bace1*		
	Drug-induction	OKA		
		Benzo[a]pyrene		
	Metal ion-induction	Al		
		Cu		
		Co		
		Fe^3+^		
		Zn^2+^		
		Ag^2+^		
		Pb^2+^		
		Ni^2+^		
		Hg^2+^		
		Mn^2+^		
		Cd^2+^		
		Cr_4_O^2-^		

PD	Gene editing	*LRRK2*	*α*-syn	[87, 156, 157, 163, 166, 168–172]
		*GBA*		
		*Parkin*		
		*DJ1*		
		*Pink1*		
		*ATP13A2*		
	Drug-induction	MPTP		
		6-OHDA	DA	
		Rotenone		
		Paraquat		
		Ziram		
		Benomyl		
		DEPe		

Epilepsy	Gene editing	*scn1a*	GABA	[190, 191, 193, 198, 200, 204, 205, 207–211]
		*KCNJ10*		
		*KCNQ3*		
			GFAP	
		*got2a*		
		*ubtor*		
		*stx1b*		
		*stxbp1*	CHD2	
		*cacna1a*		
		*CHD2*		
	Drug-induction	PTZ		
		AG		
		KA		

## Abbreviations

Δ9-THC: Δ9-Tetrahydrocannabinol
5-HT: 5-Hydroxytryptamine
6-OHDA: 6-Hydroxydopamine
AD: Alzheimer's disease
ADH: Alcohol dehydrogenase
ADHD: Attention deficit hyperactivity disorder
AEDs: Antiepileptic drugs
AG: (D, L)-Allylglycine
Al: Aluminum
ALDH: Acetaldehyde dehydrogenase
ALS: Amyotrophic lateral sclerosis
AMPA: *α*-Amino-3-hydroxy-5-methyl-4-isoxazole-propionic acid
AMPK: 5′ AMP-activated protein kinase
ARC: Activity-regulated cytoskeleton-associated protein
ASD: Autism spectrum disorder
A*β*: Amyloid-*β*
BBB: Blood-brain barrier
BMAA: *β*-N-Methylamino-L-alanine
BPA: Bisphenol A
CA: Catalase
cAMP: Cyclic adenosine 3′,5′-monophosphate
CBD: Cannabidiol
CHD2: Chromodomain helicase DNA-binding protein
CNS: Central nervous system
COMT: Catechol-O-methyltransferase
CPP: Conditioned place preference
CREB: cAMP-responsive element-binding protein
CRF: Corticotropin releasing factor
CRH: Corticotrophin-releasing hormone
CSPG: Chondroitin sulfate proteoglycan
Cu: Copper
CUMS: Chronic unpredictable mild stress
CYP2A6: Cytochrome P450 2A6
CYP2E1: Cytochrome P450 2E1
DA: Dopamine
DAT: Dopamine transporter
DALYs: Disability-adjusted life years
DEPe: Diesel exhaust particulate extracts
dpf: Days postfertilization
DPRs: Dipeptide repeats
DSM-5: Diagnostic and Statistical Manual of Mental Disorders V
DSPGs: Dermatan sulfate proteoglycans
ECM: Extracellular matrix
FA: Folic acid
FASD: Fetal alcohol spectrum disorders
FKBP5: FK506-binding protein 5
FMO: Flavin-containing monooxygenase
fMRI: Functional Magnetic Resonance Imaging
FUS: Fused in sarcoma
GABA: Gamma-aminobutyric acid
GAD: Glutamic acid decarboxylase
GFAP: Glial fibrillary acidic protein
GSH: Glutathione
GST: Glutathione-S-transferase
HA: Hyaluronic acid
Has1: Hyaluronan synthase 1
Has3: Hyaluronan synthase 3
HD: Huntington's disease
HP: Heparin
HPA: Hypothalamic-pituitary-adrenal
hpf: Hour postfertilization
HPI: Hypothalamic–pituitary–adrenal
HPT: Hypothalamic–pituitary–thyroid
HSPGs: Heparin sulfate proteoglycans
HTT: Huntingtin gene
IRS1: Insulin receptor substrate 1
JAK: Janus kinase
KA: Red alginate
KSPG: Keratan sulfate proteoglycan
LRRK2: Leucine-rich repeat protein kinase-2
mACHR: Muscarinic
MAO: Monoamine oxidase
MAP: Mitogen-activated protein
MDD: Major depression disorder
MK-801: Dizocilpine
MO: Morpholine
MPE: Moderate prenatal ethanol
MPTP: 1-Methyl-4-phenyl-1,2,3,6-tetrahydropyridine
MTOR: Mechanistic target of rapamycin
MW: Molecular-weight
N17: N-terminal 17 amino acids
NA: Noradrenaline
nAChR: Nicotine activates nicotinic
NE: Norepinephrine
NFT: Neurofibrillary tangle
NFTs: Neurofibrillary tangles
NMDA: N-Methyl-D-aspartate
NPS: New psychoactive substances
NPY: Neuropeptide Y
NTT: Novel tank test
OKA: Okadaic acid
OKR: Optokinetic response
P13K: PI3 kinase
PD: Parkinson's disease
PDK1: Pyruvate dehydrogenase lipoamide kinase isozyme 1
Pink1: Putative kinase1
PKB: Protein kinase B
PKG: Protein kinase G
PNNs: Perineuronal nets
PP2A: Protein phosphatase 2A
PPA: Propionic acid
PRD: Proline-rich domain
PSD: Postsynaptic density protein
PtdIns: Phosphatidylinositol
PTEN: Phosphatase and tensin homolog
PTSD: Posttraumatic stress disorder
PTZ: Pentylenetetrazol
QA: 3-Nitropropionic acid (3-NPA) Quinolinic acid
RHEB: Ras homolog enriched in brain
SNc: Substantia nigra pars compacta
SOD: Superoxide dismutase
SRR: Serine racemase
STK11: Serine/threonine kinase 11
STRADA: STE20-related kinase adaptor alpha
TALENS: Transcriptional activator-like effector nucleases
TDP-43: TAR-binding protein 43
TNC: Tenascin-C
TNR: TENASCIN-R
TSC: Tuberous sclerosis complex
Ub: Ubiquitin
UGT: UDP glucuronosyltransfease
VDCC: Voltage-dependent calcium channel
WHO: World Health Organization.

## Supplementary Materials

Supplementary Materials Graphical Abstract: The pathogenesis of mental illness in humans is intertwined and intricate. The causes can fall into three categories: psychoactive substances/addictive substances such as drugs side effects and addictive substances, environmental pollution and excess metal ions and congenital disease or genetic mutation. Zebrafish is a recognized animal model of mental disorders. Here, we analyze the methods used by zebrafish to model mental disorders and divided zebrafish behavioral tests of mental illness into four categories: motor and sensory functions, depression-/anxiety-like behavior, learning and memory, social behavior, to solve the indistinguishable problem of psychiatric comorbidities from zebrafish behavioral level.
