# Supplementary material for: Advances in Zebrafish as a Comprehensive Model of Mental Disorders
Source: Depress Anxiety. 2023 Jun 20;2023:6663141. doi: 10.1155/2023/6663141 (PMC11921866; doi:10.1155/2023/6663141)
Supplement: Supplementary Materials — Graphical Abstract: The pathogenesis of mental illness in humans is intertwined and intricate. The causes can fall into three categories: psychoactive substances/addictive substances such as drugs side effects and addictive substances, environmental pollution and excess metal ions and congenital disease or genetic mutation. Zebrafish is a recognized animal model of mental disorders. Here, we analyze the methods used by zebrafish to model mental disorders and divided zebrafish behavioral tests of mental illness into four categories: motor and sensory functions, depression-/anxiety-like behavior, learning and memory, social behavior, to solve the indistinguishable problem of psychiatric comorbidities from zebrafish behavioral level. [file 6663141.f1.pdf]

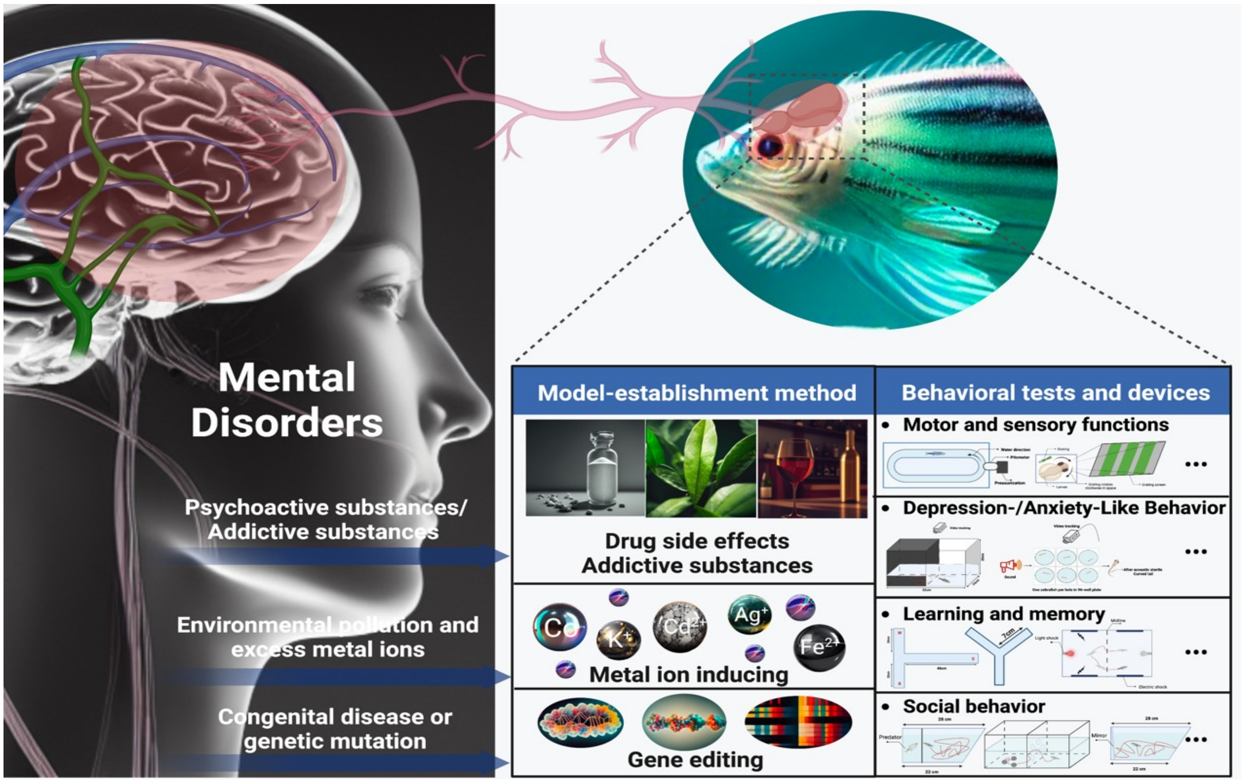

Graphical Abstract: The pathogenesis of mental illness in humans is intertwined and intricate. The causes can fall into three categories: psychoactive substances/addictive substances such as drugs side effects and addictive substances, environmental pollution and excess metal ions and congenital disease or genetic mutation. Zebrafish is a recognized animal model of mental disorders. Here, we analyze the methods used by zebrafish to model mental disorders and divided zebrafish behavioral tests of mental illness into four categories: motor and sensory functions, depression-/ anxiety-like behavior, learning and memory, social behavior, to solve the indistinguishable problem of psychiatric comorbidities from zebrafish behavioral level.
